# Systems analysis of apoptotic priming in ovarian cancer identifies vulnerabilities and predictors of drug response

**DOI:** 10.1038/s41467-017-00263-7

**Published:** 2017-08-28

**Authors:** Ioannis K. Zervantonakis, Claudia Iavarone, Hsing-Yu Chen, Laura M. Selfors, Sangeetha Palakurthi, Joyce F. Liu, Ronny Drapkin, Ursula Matulonis, Joel D. Leverson, Deepak Sampath, Gordon B. Mills, Joan S. Brugge

**Affiliations:** 1 Department of Cell Biology, Ludwig Center at Harvard, Harvard Medical School, Boston, MA 02115 USA; 2 Belfer Center for Applied Cancer Research, Dana Farber Cancer Institute, Boston, MA 02115 USA; 3 Department of Medical Oncology, Dana Farber Cancer Institute, Boston, MA 02115 USA; 4 Penn Ovarian Cancer Research Center, Department of Obstetrics and Gynecology, University of Pennsylvania School of Medicine, Philadelphia, PA 19104 USA; 5 Oncology Development, AbbVie, Inc, North Chicago, IL 60064 USA; 6 Translational Oncology, Genentech, South San Francisco, CA 94080 USA; 7 Department of Systems Biology, MD Anderson Cancer Center, Houston, TX 77030 USA

## Abstract

The lack of effective chemotherapies for high-grade serous ovarian cancers (HGS-OvCa) has motivated a search for alternative treatment strategies. Here, we present an unbiased systems-approach to interrogate a panel of 14 well-annotated HGS-OvCa patient-derived xenografts for sensitivity to PI3K and PI3K/mTOR inhibitors and uncover cell death vulnerabilities. Proteomic analysis reveals that PI3K/mTOR inhibition in HGS-OvCa patient-derived xenografts induces both pro-apoptotic and anti-apoptotic signaling responses that limit cell killing, but also primes cells for inhibitors of anti-apoptotic proteins. In-depth quantitative analysis of BCL-2 family proteins and other apoptotic regulators, together with computational modeling and selective anti-apoptotic protein inhibitors, uncovers new mechanistic details about apoptotic regulators that are predictive of drug sensitivity (BIM, caspase-3, BCL-X_L_) and resistance (MCL-1, XIAP). Our systems-approach presents a strategy for systematic analysis of the mechanisms that limit effective tumor cell killing and the identification of apoptotic vulnerabilities to overcome drug resistance in ovarian and other cancers.

## Abstract (web summary)

High-grade serous ovarian cancers (HGS-OvCa) frequently develop chemotherapy resistance. Here, the authors through a systematic analysis of proteomic and drug response data of 14 HGS-OvCa PDXs demonstrate that targeting apoptosis regulators can improve response of these tumors to inhibitors of the PI3K/mTOR pathway.

## Introduction

High-grade serous ovarian cancer (HGS-OvCa) accounts for 70–80% of ovarian cancer deaths and, despite optimized surgery and chemotherapy protocols, treatment resistance ultimately emerges in most cases^1^. Therefore, there is an urgent need to develop new therapies to improve patient outcomes^2^. Although therapeutically actionable recurrent point mutations are uncommon in HGS-OvCa, genomic and proteomic characterization of primary tumors have uncovered commonly deregulated signaling pathways that represent attractive targets for therapeutic intervention^3–5^. In particular, multiple components of the phosphoinositide 3-kinase/AKT/mammalian target of rapamycin (PI3K/AKT/mTOR) pathway are genetically altered in HGS-OvCa tumors (PTEN^3^ copy number loss, AKT^3^ and PIK3CA^3, 6^ copy number amplification) and there is evidence for pathway activation based on increased phosphorylation of key nodes (phospho-AKT^7^, phospho-GSK3^7^, phospho-PRAS40^8^, phospho-p70^RSK^ ^9^, an phospho-S6^8^). High PI3K/AKT/mTOR pathway activity in HGS-OvCa tumors has been associated with decreased patient survival^10–12^ and therefore represents an important therapeutic target. To date, clinical evaluation of multiple drugs targeting different nodes of the PI3K/AKT/mTOR pathway has revealed limited efficacy as single-agents^13^ and multiple resistance mechanisms have been identified^14, 15^. The identification of drugs that optimally synergize with PI3K/AKT/mTOR inhibition is critical for effective targeting of HGS-OvCa tumors with PI3K/AKT/mTOR pathway activation^15, 16^.

Preclinical studies using established ovarian cancer cell lines have described combinations of PI3K inhibitors with chemotherapy^17^ and various agents targeting the RAS/ERK pathway^18^, EGFR^19^, mTOR^20^, and BCL-2-family proteins^21, 22^. However, genomic^23^ and tumor xenograft studies^24, 25^ have called into question the suitability of many commonly used ovarian cancer cell lines as models of HGS-OvCa. Patient-derived xenograft (PDX) models, on the other hand, represent a more clinically relevant tool for studying drug treatment efficacy, as they have been shown to mirror clinical responses and recapitulate resistance mechanisms seen in patients^26, 27^ and retain the genetic heterogeneity of human tumors more faithfully than established cell lines^28–30^. Given their genomic heterogeneity, PDX models may also be more relevant for biomarker discovery^31^ to enable appropriate patient selection, an important consideration given that PI3K/AKT/mTOR-therapies in combination with other targeted agents are currently under clinical evaluation^16^. Systems biology approaches to cancer offer a framework to integrate these heterogeneous PDX responses with mathematical models to enhance our understanding of resistance mechanisms and to design effective combination therapies linked to biomarkers able to identify patients most likely to benefit^32, 33^.

Here, we present an integrated systems biology approach combining computational, proteomic and drug response profiling to identify apoptotic vulnerabilities and effectively kill tumor cells in HGS-OvCa PDX models. These PDX models exhibit heterogeneous PI3K/AKT/mTOR pathway activation at the protein level. We show that, despite diverse signaling responses in the PDX models, PI3K/mTOR inhibition results in elevated apoptotic protein levels (i.e., apoptotic priming) across all models, thus presenting a potentially exploitable therapeutic vulnerability. We exploit this vulnerability by combined inhibition of the PI3K/AKT/mTOR axis and BCL-2/BCL-X_L_; this combination treatment induces cell death in short-term in vitro cultures and in orthotopic PDX xenografts in vivo. In-depth analysis of BCL-2 family proteins and other apoptotic regulators in response to PI3K/mTOR pathway blockade identifies BIM, caspase-3, BCL-X_L_, XIAP, and MCL-1 as critical players in ovarian cancer cell survival. Our study reveals specific apoptotic vulnerabilities in a heterogeneous panel of patient-derived ovarian cancer cells in order to rationally identify effective combination therapies and candidate response biomarkers that exploit these vulnerabilities.

## Results

### PI3K/mTOR pathway activation and single-agent drug sensitivity

**Fig. 1:**
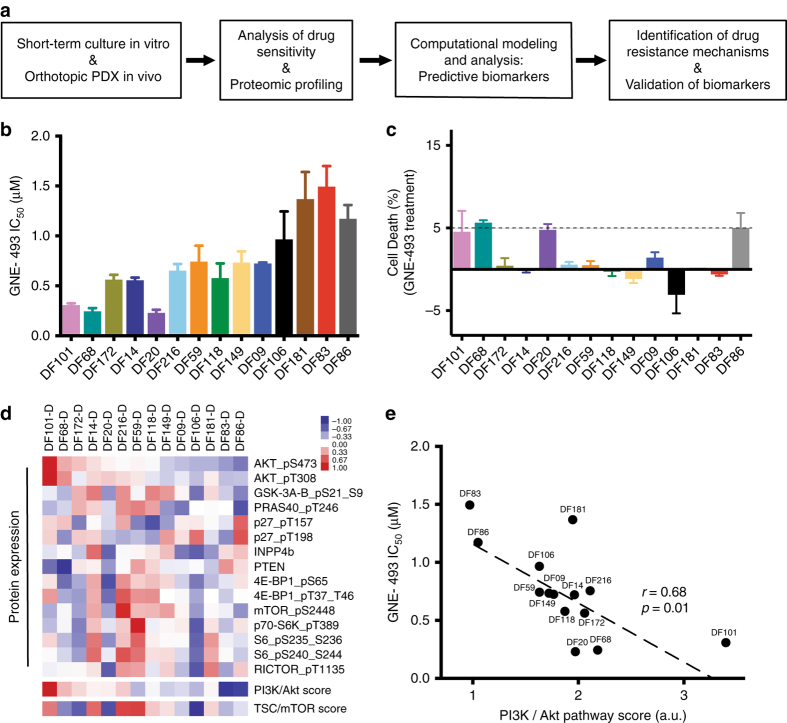
Analysis of PI3K/AKT/mTOR pathway activation at the protein level and sensitivity to PI3K/mTOR inhibition. **a** A systems approach strategy using a panel of 14 HGS-OvCa PDX and integrated drug profiling/proteomics to identify drug resistance mechanisms and validate response biomarkers. **b** PI3K/mTOR inhibitor (GNE-493) IC_50_ values ranked according to PI3K/AKT activation status (phospho-AKT^S473^ protein levels). Data is derived from three independent experiments and error bars denote SEM. **c** cell death after PI3K/mTOR treatment (GNE-493 0.3 μM, 96 h) using laser scanning cytometry. Data is representative of three independent experiments. Error bars represent SEM for *n* = 6 replicate wells. **d** Heatmap showing protein levels (median-centered) of PI3K/AKT/mTOR pathway targets for PDX samples under short-term in vitro culture conditions. Samples ranked according to PI3K/AKT activation status (data is representative of two independent experiments). **e** Correlation analysis of GNE-493 IC_50_ values with PI3K/AKT pathway score (Pearson *r* = 0.68, *p* = 0.01)

We developed an unbiased systems biology framework that combines drug profiling with proteomics to identify effective drug combinations and response biomarkers, and study resistance mechanisms using a well-annotated panel of 14 HGS-OvCa PDX models (Fig. 1a). These PDX models were generated from ascites/pleural effusions of patients with advanced ovarian cancer and faithfully recapitulate the histology and molecular characteristics of the original tumor^34^. This PDX panel exhibits significant molecular and phenotypic diversity (e.g., BRCA germline mutations, DNA copy number alterations, in vivo growth rates and drug responses) and presents a clinically relevant resource to identify potential therapeutic targets and assess treatment efficacy.

To analyze the responsiveness of PDX models to PI3K/mTOR pathway inhibition and comprehensively measure proteomic profiles, we established a pipeline to process ascites samples from the PDX models in short term in vitro cultures. We engineered these PDX models to express an mCherry and luciferase reporter, to monitor cell numbers in vitro and tumor growth in vivo (Supplementary Fig. 1A, B). Given that the PI3K/AKT/mTOR pathway is commonly deregulated in human ovarian cancer^3^ and several of the PDX models contain deletions or amplifications in key pathway nodes^34^ (Supplementary Fig. 1C), we chose to initially examine the efficacy of the PI3K/mTOR inhibitor GNE-493^35^. Treatment with GNE-493 resulted in a dose-dependent reduction in cell numbers across all PDX models (Supplementary Fig. 1D), with a 7-fold range in GNE-493 IC_50_ values, from 0.2 ± 0.03 μM to 1.5 ± 0.2 μM (Fig. 1b). PI3K/mTOR inhibition elicited predominantly cytostatic effects, with a low level of cell death induction (5%) in four models (Fig. 1c).

To assess relative PI3K/AKT/mTOR pathway activation at baseline, we performed reverse phase protein arrays (RPPA) that monitor multiple signaling nodes of this pathway (short term in vitro cultures: Fig. 1d and in vivo PDX: Supplementary Fig. 1E). We used a scoring method^8^ that evaluates the extent of pathway activation by assessing multiple phosphoprotein activation sites (e.g., phospho-AKT^308,473^), protein targets (e.g., phospho-GSK-3^21/9^, phospho-PRAS40^246^) and pathway inhibitors (e.g., PTEN, INPP4B). The PDX models exhibited wide variation in the extent of pathway activation at the protein level and ranking of the PDX models according to PI3K/AKT pathway activation levels was comparable between short-term in vitro cultures and xenografts grown orthotopically (Supplementary Fig. 1E). These results provide supportive evidence for our strategy to initially screen in short term in vitro cultures.

Next, we examined the correlation of PI3K/AKT and TSC/mTOR pathway scores with GNE-493 IC_50_ values and found a significant correlation only for the PI3K/AKT score (Fig. 1e, Supplementary Table 1, Pearson correlation coefficient (*r*), *r* = 0.68, *p* = 0.01). We also investigated the correlation of individual signaling nodes with drug sensitivity (Supplementary Table 2) and found that GNE-493 sensitivity correlated with high levels of phospho-AKT^473^ and phospho-AKT^308^ (Pearson *r* = 0.75, *p* = 0.002 and *r* = 0.60, *p* = 0.02, respectively), and with low levels of PTEN (Pearson *r* = 0.61, *p* = 0.02). HGS-OvCa PDX models, therefore, display a predominantly cytostatic response to PI3K/mTOR inhibition, with varying drug sensitivity that correlates with baseline PI3K/AKT pathway activity.

### PI3K/mTOR-inhibition induces an elevated apoptosis signature

**Fig. 2:**
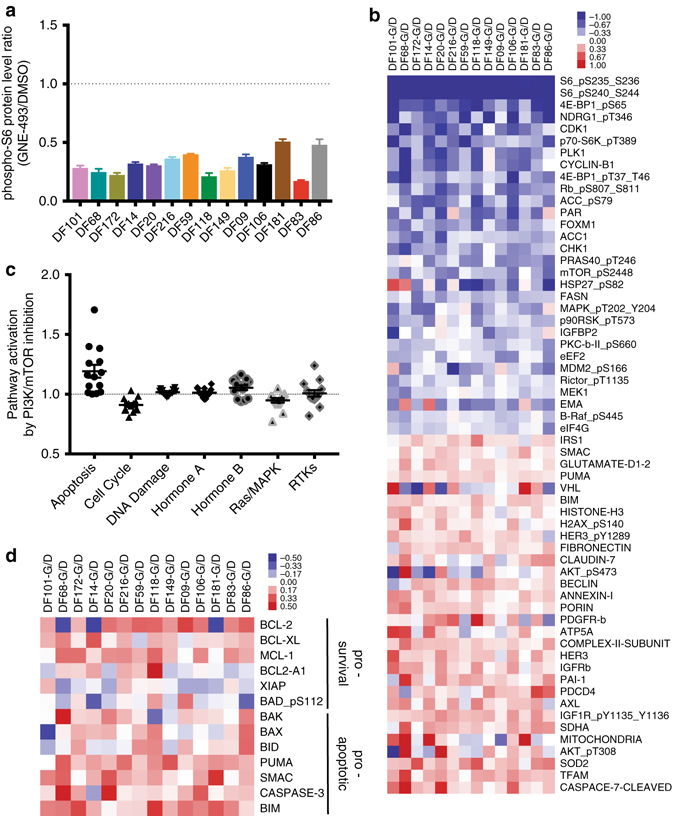
Rewiring of multiple pathways after PI3K/mTOR inhibition and upregulation of pro-apoptotic and anti-apoptotic proteins in all PDX samples. **a** Analysis of phospho-S6 protein levels after treatment with 0.5 μM GNE-493 (48 h). Mean values for ratio of GNE-493 over DMSO protein levels for each PDX model across three replicate samples. **b** Heatmap showing proteins exhibiting the largest fold increases (red) or decreases (blue) relative to baseline after GNE-493 treatment across all 14 PDX samples. Protein lysates from PDX cells treated with 0.5 μM GNE-493 for 48 h were analyzed by RPPA and all signals from all samples were normalized to DMSO control. Data is representative of two independent experiments and represents the mean log2 transformed value for *n* = 3 replicates. **c** Analysis of pathway scores (mean value for the ratio of GNE-493 over DMSO score values, data is derived from **b**). **d** Heatmap of anti-apoptotic and pro-apoptotic relative protein levels after GNE-493 treatment (data represents the ratio of protein levels of GNE-493 treatment to DMSO (G/D) and is derived from **b**)

Given our interest in identifying candidate vulnerabilities for the rational design of PI3K/mTOR-based combination therapies, we examined the activation of a broad range of signaling pathways in our PDX models after treatment with GNE-493 using RPPA. Global analysis of 288 proteins and phosphoproteins representing major signaling pathways identified those proteins that were significantly altered (*p* < 0.05, Student’s *t*-test) in at least one PDX model (Supplementary Data 1). To visualize those proteins with the largest fold-changes following treatment with GNE-493 in short-term in vitro cultures, we created a heatmap of the proteins exhibiting the largest fold increases (red) or decreases (blue) relative to baseline. Phosphoprotein levels of downstream targets of the PI3K/mTOR pathway (phospho-PRAS40, phospho-NDRG1, phospho-S6, phospho-4E-BP1, and phospho-p70^RSK^) were reduced by 20–70%, indicating effective PI3K/mTOR pathway inhibition across all PDX models (Fig. 2a, b and Supplementary Fig. 2A). The strong reduction in PI3K/mTOR substrates and the lack of correlation between GNE-493 sensitivity (IC_50_) and the fold-change in phospho-S6 protein levels after PI3K/mTOR inhibition (Supplementary Fig. 2B), suggested that differences in the sensitivity to GNE493 are not strongly influenced by differences in pathway inactivation. Consistent with the measured cytostatic effects (Fig. 1c), cell proliferation markers (Cyclin B1, pRB, CDK1) were also largely reduced (Fig. 2b). The proteins with the most induced levels included pro-survival receptor tyrosine kinases (RTKs: phospho-IGF1R, phospho-HER3) and mitochondrial proteins (SOD2, TFAM, GLUD1, SDHA)^36^.

To systematically identify commonly altered signaling pathways across the 14 PDX models, we computed pathway scores for seven major pathways^8^ (Fig. 2c) for which selective inhibitors exist and are in the final stages of clinical development (Supplementary Table 3 and 4). Consistent with the decrease of individual cell proliferation markers, we found that the cell cycle pathway score was reduced in 13/14 PDX models (Supplementary Fig. 2C). The RAS/ERK pathway activity was also reduced by PI3K/mTOR inhibition, indicative of pathway cross-activation as previously described in breast cancer cells^37^. PI3K/mTOR inhibition induced a small increase in DNA damage score as previously reported in established ovarian cancer cell line studies^38^.

The apoptosis pathway score was increased in all 14 PDX models and exhibited the highest percent-change (up to 71%, *p* = 0.002, Student’s *t*-test) compared to the other pathways (Fig. 2c). This score includes contributions from both pro-apoptotic BH3-only proteins (BIM, BID, PUMA) and multi-domain proteins (BAX and BAK), as well as from anti-apoptotic proteins (BCL-2, BCL-X_L_, BCL-2A1, and MCL-1). The majority of these proteins exhibited an increasing trend (Fig. 2d and Supplementary Fig. 2D). The balance between pro-apoptotic and anti-apoptotic proteins is critical in determining whether cells undergo apoptosis; tumor cells with high pro-apoptotic protein levels are primed for cell death (i.e., apoptotic priming) once anti-apoptotic proteins are inhibited^39, 40^. Our findings suggest that the ovarian PDX cancer cells maintain a balance between pro- and anti-apoptotic proteins after GNE-493 treatment that could be exploited using combination therapies that inhibit the anti-apoptotic proteins, thereby enhancing the efficacy of PI3K-directed therapies.

### Synergistic PI3K/mTOR and BCL-2/BCL-X_L_ drug combination

**Fig. 3:**
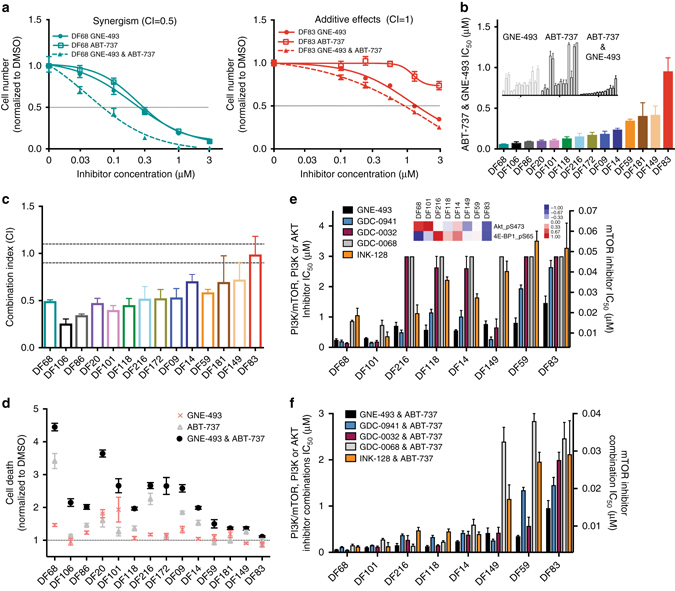
Inhibition of PI3K/mTOR and BCL-2/BCL-X_L_ is synergistic and induces significant cell death in vitro. **a** Representative examples of synergistic (DF68) and additive effects (DF83). Data is representative of three independent experiments and error bars represent SEM (*n* = 6 wells). **b** Ranking of PDX models based on their IC_50_ values for the GNE-493 & ABT-737 combination. Inset shows IC_50_ values for single-agent and drug combination. Data is derived from experiment in **a** and Supplementary Fig. 3A, B. **c** Combination indices computed using the Chou–Talalay analysis for all 14 PDX samples. Values are averages across three independent experiments and error bars represent SEM. **d** Analysis of cell death induced by single agent and combined GNE-493 and ABT-737 (compared to DMSO) 96 h after drug exposure (0.3 μM of each drug) using laser scanning cytometry. Data is representative of three independent experiments and error bars are SEM (*n* = 6 wells). **e**, **f** IC_50_ values for GDC-0032 (PI3K-α/γ/δ inhibitor), GDC-0941 (pan-PI3K inhibitor), INK-128 (mTORC1/2 inhibitor) and GDC-0068 (AKT inhibitor) as single agents **e** and in combination **f** with ABT-737 compared to the GNE-493 and ABT-737 combination in a panel of eight PDX models. Heatmap shows phospho-AKT and phospho-4EBP1 protein levels (data derived from Fig. 1d). Data is derived from Supplementary Fig. 4 and represents an average of three independent experiments (error bars represent SEM, *n* = 3)

To evaluate whether inhibition of anti-apoptotic proteins could enhance the efficacy of GNE-493, we examined the sensitivity of the PDX models to the combination of the dual BCL-2/BCL-X_L_ inhibitor ABT-737 and GNE-493 using the Chou–Talalay method to assess drug synergy (Combination Index, CI)^41^. Figure 3a depicts an example of a PDX model that shows synergistic effects (DF68, CI ~ 0.5) and one that shows additive effects (DF83, CI ~ 1) of the drug combination. In multiple PDX models, the IC_50_ values of the GNE-493 and ABT-737 drug combination were in the nanomolar range (Fig. 3b) and lower than those for either single-agent treatment across all 14 PDX models (Supplementary Fig. 3A–C). The GNE-493 and ABT-737 combination was synergistic for the majority of the PDX models (Fig. 3c).

We next compared the effects of the drug combination versus single-agent treatment on cell death. Single-agent treatment with ABT-737 resulted in a small increase in cell death in the majority of the PDX samples compared to the control (less than 2-fold in 12/14 models) (Fig. 3d and Supplementary Fig. 3D). In contrast to the cytostatic effects of the PI3K/mTOR inhibitor, the drug combination increased tumor cell death in the majority of the PDX models. The highest fold-changes in cell death (4.3-fold and 4.7-fold) were observed in the models with the lowest IC_50_ values to the drug combination (DF20 and DF68); however, those models with the highest IC_50_ values (DF181, DF149, DF59, and DF83) showed minimal increase in cell death.

The synergy observed for the GNE-493 and ABT-737 combination prompted us to examine whether selective PI3K/AKT/mTOR pathway inhibitors could exert similar effects. We assessed the effects of the AKT inhibitor ipatasertib (GDC-0068), the PI3K-β isoform-sparing PI3K inhibitor tasesilib (GDC-0032), the pan-PI3K inhibitor pictilisib (GDC-0941), and the mTORC1/2 inhibitor sapanisertib (INK-128) in PDX models with a range of GNE-493 sensitivities. First, we examined the effects of these inhibitors as single-agents. Overall, the ranking of the PDX models was similar between the selective inhibitors and the dual PI3K/mTOR inhibitor (Fig. 3e and Supplementary Fig. 4). PDX models with medium phospho-AKT, but high phospho-4EBP1 protein levels (DF216, DF118, DF14, Fig. 3e) were sensitive to mTOR inhibition either by INK-128 or GNE-493 and responded less well to selective PI3K or AKT inhibition by GDC-0032, GDC-0941 and GDC-0068. These results highlight the potential to individualize PI3K/AKT/mTOR treatment based on the expression of PI3K or mTOR pathway targets.

We also assessed the effects of selective PI3K/AKT/mTOR inhibitors in combination with BCL-2/BCL-X_L_ inhibition. We found that the GDC-0068 & ABT-737 combination had similar effects as the GNE-493 & ABT-737 combination in the five most sensitive PDX models (DF68, DF101, DF118, DF216, DF14) but was less effective in the three least sensitive PDX models (DF59, DF149, DF83) (Fig. 3f and Supplementary Fig. 4). The IC_50_ values for the two PI3K and BCL-2/XL inhibitor combinations (GDC-0032 & ABT-737 and GDC-0941 & ABT-737) were similar to each other across the PDX models. Compared with the GNE-493 & ABT-737 combination, the GDC-0032 and GDC-0941 combination with ABT-737 exhibited similar effects in 6/8 PDX models, including the five most sensitive PDX models described above and model DF149 with intermediate sensitivity. In the two least sensitive PDX models (DF59 and DF83) higher doses of the GDC-0032 & ABT-737 and GDC-0941 & ABT-737 combinations were required to match the growth-inhibition of the GNE-493 & ABT-737 combination. The INK-128 & ABT-737 combination was also effective in the five most sensitive PDX models. Taken together, these results demonstrate that AKT, PI3K, or mTOR blockade in combination with ABT-737 is as effective as dual PI3K/mTOR inhibition in combination with ABT-737 in the sensitive PDX models, and that selective mTOR inhibitors are effective for PDX models with high phospho-4EBP1 protein expression.

**Fig. 4:**
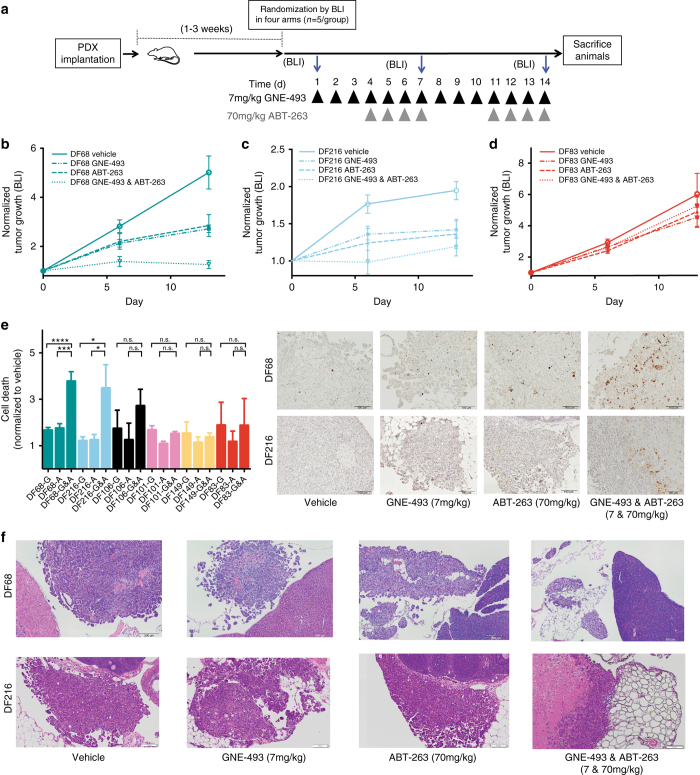
Combination of PI3K/mTOR and BCL-2/BCL-X_L_ inhibition enhances the activity of single-agent treatment in vivo and increases cell death in a subset of PDX samples. **a** Combination treatment dosing schedule. **b**, **c**, **d** Tumor growth curve (determined by bioluminescence imaging (BLI)) of three PDX models (DF68, DF216, DF83) that exhibit different sensitivities to the PI3K/mTOR (7 mg kg^−1^ GNE-493) and BCL-2/BCL-X_L_ (70 mg kg^−1^ ABT-263) drug combination. *n* = 5 per treatment arm. Error bars represent SEM. **e** Quantification of tumor cell death by cleaved-caspase 3 (cc3) immunohistochemistry (IHC) and representative cc3 images in tumors (scale bar 100 μm). Error bars represent SEM across *n* = 30 locations from three independent animals (Kruskal–Wallis test: *p < 0.05, **p < 0.01, ***p < 0.001, ****p < 10^−4^). **f** Representative histological images of solid tumor implants in the abdominal cavity in two responsive PDX samples (DF68 and DF216). Scale bar 200 μm

Next, we examined whether the relative efficacy of the combined inhibition of PI3K/mTOR (GNE-493) and BCL-2/BCL-X_L_ (navitoclax, ABT-263) in vitro is predictive of in vivo responses. Six PDX models were selected based on the IC_50_ values of the drug combination to capture a range of sensitivities. We designed a pulsed dosing schedule to minimize ABT-263 induced thrombocytopenia, while maintaining effective pathway blockade (Fig. 4a and Supplementary Fig. 5A). Furthermore, using this pulsed dosing schedule, platelets recovered to pre-treatment levels 3 days after cessation of treatment providing an approach to ameliorate toxicity (Supplementary Fig. 5B). Single-agent treatment with GNE-493 reduced tumor growth in four models (DF68, DF106, DF101, and DF216), with the best response of 46 ± 3% tumor growth inhibition (DF68), and the poorest at 1 ± 10% tumor growth inhibition (DF149, Supplementary Fig. 5C). Single agent ABT-263 treatment also reduced tumor growth in most models, with a maximum inhibitory response at 43 ± 9% (DF68) and the weakest at 18 ± 16% (DF83, Supplementary Fig. 5C). Combined GNE-493 and ABT-263 treatment reduced tumor growth to varying degrees compared to vehicle and each agent alone (Fig. 4b–d and Supplementary Fig. 5D–F). For example, DF68, which exhibited the lowest IC_50_ value in vitro, showed the best response with 75 ± 4% inhibition (Fig. 4b) wheras DF83, which exhibited the highest IC_50_ value in vitro, showed a minimal response with 12 ± 11% inhibition (Fig. 4d). We also assessed the relationship between tumor growth inhibition monitoring using bioluminescence imaging (BLI) and tumor burden measurements using tumor ascites volume and found a strong correlation (Pearson *r* = 0.64, *p* = 0.004, Supplementary Fig. 5G). Ranking of the six PDX models according to their sensitivity to the drug combination was similar in vitro and in vivo (Supplementary Fig. 5H). In addition, ranking of all PDX models according to expression of BCL-2 family proteins was similar in short term in vitro cultures and in vivo (Supplementary Fig. 5I). All treatments were well tolerated as indicated by minimal changes in animal body weights (Supplementary Fig. 5J).

To evaluate whether the drug combination induced tumor cell death in vivo, we performed histological analyses of solid tumors and ascites cancer cells. Consistent with the observed range of responses in tumor growth inhibition, we found varying degrees of induction of cleaved-caspase 3-positive cells by the drug combination compared to either single-agent treatment (Fig. 4e and Supplementary Fig. 6). The two models that showed the best efficacy (DF216 and DF68) showed significantly higher cell death induction for the drug combination compared to either single-agent treatment (Fig. 4e, 3.5-fold, *p* = 0.02, and 3.8-fold, *p* = 0.001, Kruskal–Wallis test). Histological analysis revealed that the drug combination induced marked necrosis in the responsive PDX model DF216 and significant reduction in tumor size of the most sensitive PDX model DF68 compared to the vehicle or either single-agent treatment arm (Fig. 4f and Supplementary Fig. 6). Collectively, these data demonstrate that the PI3K/mTOR and BCL-2/BCL-X_L_ drug combination is tolerated in orthotopic HGS-OvCa PDX models and, in a subset of these PDXs, can induce a strong antitumor effect.

### Computational modeling identifies drug response predictors

**Fig. 5:**
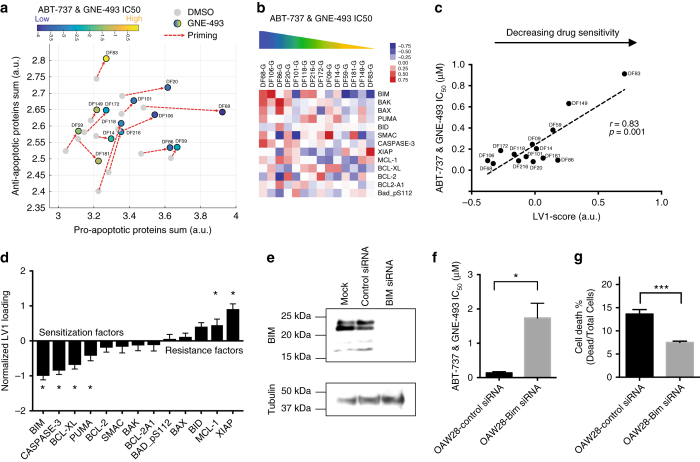
Computational modeling identifies predictors of sensitivity and resistance to combined PI3K/mTOR and BCL-2/BCL-X_L_ inhibition. **a** Relationship between apoptotic priming (vector-plot: shows change induced by GNE-493 treatment) and GNE-493 and ABT-737 sensitivity (IC_50_ values) **b** Heatmap of apoptosis-regulating proteins (median-centered) after GNE-493 treatment (48 h, 0.5 μM). **c** Partial least-squares regression (PLSR) analysis identifies a latent variable LV1 (LV1 score) that correlates with drug sensitivity. **d** Ranking of the contribution of each apoptosis-regulating protein in LV1. Mean ± SD for each protein from a leave-one-out cross validation analysis. Proteins with regression coefficients different than zero are marked with an asterisk. **e** BIM siRNA knockdown reduces BIM protein levels (96 h after knockdown) in OAW28. **f** Analysis of GNE-493 and ABT-737 IC_50_ value after BIM siRNA knockdown in OAW28. **g** BIM siRNA knockdown rescues cell death (laser scanning cytometry measurements) induced by the GNE-493 and ABT-737 drug combination in OAW28. Errors are SEM for *n* = 4 replicate wells and results are representative of two independent experiments. (Two-tailed student *t*-test: *p < 0.05, ***p < 0.001)

The range of responses to the PI3K/mTOR and BCL-2/BCL-X_L_ combination in the PDX models offers a valuable resource to discover markers of drug sensitivity and resistance mechanisms. Given that the apoptosis score exhibited the largest increase compared to other pathways (Fig. 2c), we examined its relationship to sensitivity to PI3K/mTOR and BCL-2/BCL-X_L_ combination treatment and found that it is predictive of drug sensitivity (Fig. 5a, Supplementary Fig. 7A and Supplementary Table 5). Because the apoptotic score includes contributions from multiple pro- and anti-apoptotic proteins, we performed partial least-squares regression (PLSR) analysis to assess the relative importance of each pro-apoptotic and anti-apoptotic protein in predicting drug sensitivity using RPPA measurements following treatment with GNE-493 (Fig. 5b). The first latent variable (LV1) of the PLSR model (LV1-score: weighted sum of the apoptotic proteins) correlated significantly (Pearson *r* = 0.83, *p* = 0.001) with the magnitude of the IC_50_ to PI3K/mTOR and BCL-2/BCL-X_L_ inhibition (Fig. 5c and Supplementary Fig. 7B). Analysis of the regression coefficients of the proteins composing LV1 showed that coefficients for BIM, caspase-3, BCL-X_L_, PUMA, MCL-1, and XIAP were significantly different than zero (Fig. 5d). Specifically, BIM, caspase-3, BCL-X_L_ and PUMA had a negative coefficient indicating that high protein levels predict low IC_50_ values (drug sensitivity), while MCL-1 and XIAP had a positive coefficient correlating with high IC_50_ values (drug resistance).

Based on this multivariate analysis, BIM was the strongest individual predictor of drug sensitivity (Fig. 5d). Higher BIM levels also correlated with increased drug sensitivity using univariate analysis (Supplementary Fig. 7C, Pearson *r* = 0.59, *p* = 0.03). BIM protein expression levels from the RPPA analysis in the PDX models were validated by western blotting (Supplementary Fig. 7D). We also evaluated the power of BIM as a predictive biomarker of response to GNE-493 and ABT-737 combination treatment in an independent set of seven established ovarian cancer cell lines exhibiting diverse BIM protein levels (Supplementary Fig. 7E). Dose-response analysis of the GNE-493 and ABT-737 drug combination revealed a one-log range of sensitivity (Supplementary Fig. 7F). Similar to our findings in the PDX models, BIM protein levels correlated significantly with drug sensitivity (GNE-493 and ABT-737 IC_50_) in the ovarian cancer cell lines (Pearson *r* = 0.7, *p* = 0.004; Fig. 5g). To investigate the functional role of BIM in drug responsiveness, we performed siRNA knockdown experiments in OAW28 cells, that express high BIM protein levels and exhibit a low GNE-493 and ABT-737 IC_50_ (Supplementary Fig. 7E–F). siRNA knockdown effectively decreased BIM protein levels (Fig. 5e) and was associated with desensitization to the GNE-493 and ABT-737 combination, with a 13-fold increase (*p* = 0.01, Student’s *t*-test) in the IC_50_ value (Fig. 5f and Supplementary Fig. 7H). Attenuation of BIM expression also significantly decreased cell death in response to the GNE-493 and ABT-737 combination (by 45%, *p* = 0.001, Student’s *t*-test) (Fig. 5g). Taken together, these data indicate that reduction of BIM protein levels can desensitize ovarian cancer cells to the PI3K/mTOR and BCL-2/BCL-X_L_ drug combination and further highlight the critical role of BIM levels in predicting drug sensitivity.

**Fig. 6:**
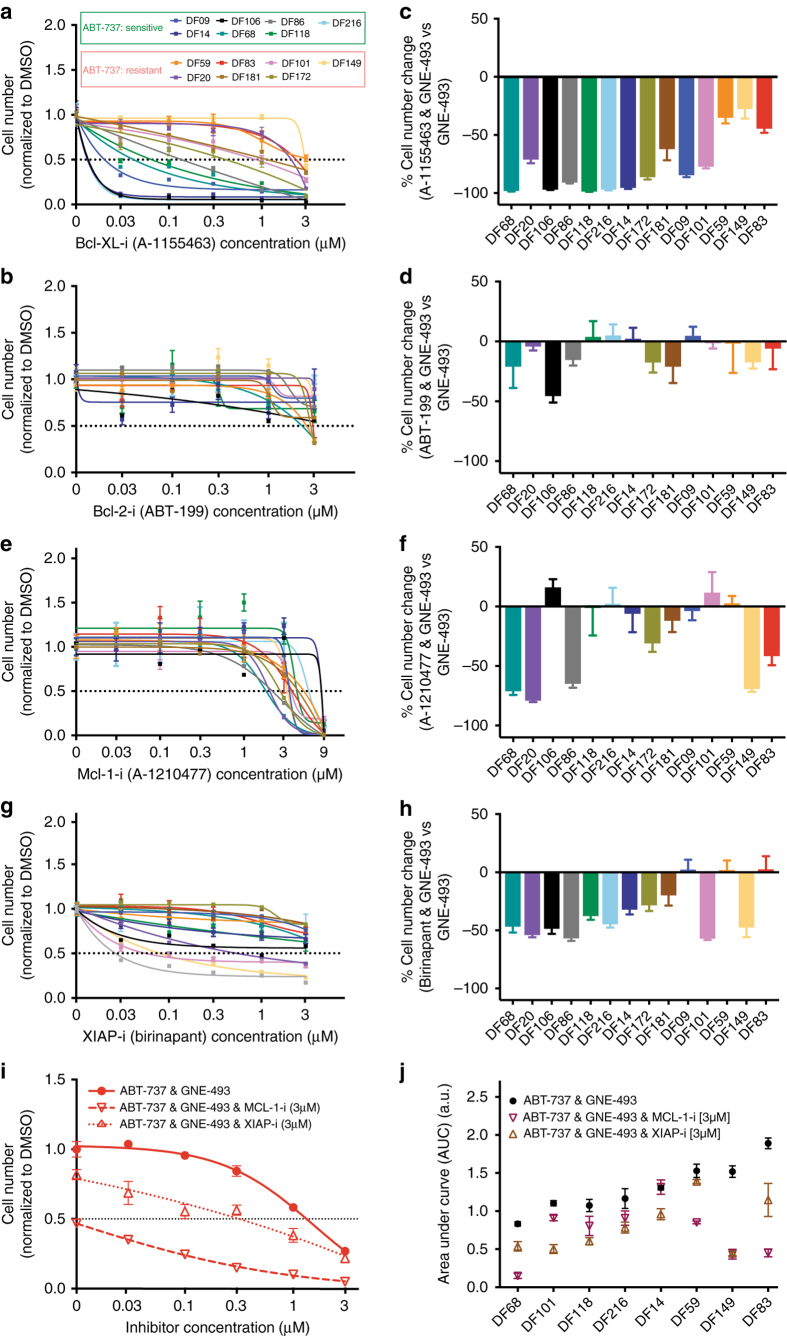
Analysis of apoptotic vulnerabilities **a**–**h** Cells were incubated with increasing concentrations of the **a** BCL-X_L_-specific inhibitor, **b** BCL-2-specific inhibitor, **e** MCL-1 and **g** XIAP-specific inhibitor for 96 h. Error bars are SEM (*n* = 6 wells) and data is representative of three independent experiments. BCL-X_L_ inhibitor **c** combined with PI3K/mTOR compared to BCL-2 **d** or MCL-1 **f** or XIAP **h** combinations. BCL-2-specific inhibitor and BCL-X_L_-specific inhibitor combinations with PI3K/mTOR inhibitor are dosed at 1 μM, while MCL-1 and XIAP combination are dosed at 3 μM. Data is representative of three independent experiments. **i** Effects of triple MCL-1 (dashed curve) or XIAP drug combination (dotted curve) combined with ABT-737 and GNE-493 for a resistant (DF83) PDX model. MCL-1 and XIAP inhibitors are at a constant concentration of 3 μM, while ABT-737 and GNE-493 increase from 0.03-3 μM. **j** Sensitivities (area under the curve values) for the triple combination therapies highlight the increased potency of MCL-1 compared to XIAP inhibition and the intertumoral heterogeneity. Data represents average from three independent experiments (error bars are SEM, *n* = 3) and is derived from experiment in **i** and Supplementary Fig. 9A

The PLSR analysis indicated that caspase-3, a downstream effector of apoptosis, is also a sensitization factor for the GNE-493 and ABT-737 drug combination. We also performed univariate regression analysis and found that high caspase-3 protein levels correlate with low IC_50_ values to GNE-493 and ABT-737 drug combination (Pearson *r* = 0.64, *p* = 0.01, Supplementary Fig. 7i). High BCL-X_L_ levels were also predicted to correlate with sensitivity to the drug combination (Fig. 5d). Since ABT-737 targets both BCL-2 and BCL-X_L_, we investigated the contribution of both of these anti-apoptotic proteins to tumor cell viability by using specific inhibitors as single agents and in combination with GNE-493. We found that treatment with the BCL-X_L_-specific inhibitor (A-1155463) mirrored the effects of ABT-737 (Fig. 6a), while treatment with the BCL-2-specific inhibitor (ABT-199, venetoclax) had no effect on any of the PDX models at concentrations in which ABT-199 selectively antagonizes BCL-2 (<1 μM, Fig. 6b), suggesting that BCL-X_L_ contributes much more significantly to viability than BCL-2 in these models. This conclusion was further supported by the evidence that combination of GNE-493 and A-1155463 led to significant sensitization of the ovarian cancer cells, with up to 98–99% inhibition for the most sensitive models (Fig. 6c, Supplementary Fig. 8A) and the potency of this combination was predicted by BCL-X_L_ protein levels (Supplementary Fig. 8B). In contrast, addition of BCL-2 inhibition to GNE-493 did not enhance its efficacy (Fig. 6d, Supplementary Fig. 8C), providing further evidence that BCL-2 does not represent an apoptotic vulnerability in these HGS-OvCa PDX models.

### Inhibition of MCL-1 or XIAP overcomes drug resistance

We also investigated the contribution of MCL-1 and XIAP, a caspase antagonist, to treatment responses since these two anti-apoptotic proteins were identified by computational modeling to predict resistance to the combination of GNE-493 and ABT-737 (Fig. 5d). We first examined the effects of an MCL-1 inhibitor (A-1210477) and a XIAP/cIAP inhibitor (birinapant) as single-agents or in combination with GNE-493. Most of the PDX models exhibited IC_50_ values for A-1210477 in the 2–6 μM range, similar to the concentration required for half-maximal dissociation of MCL-1–BIM complexes (Fig. 6e)^42^. MCL-1 inhibition in combination with GNE-493 distinguished two response classes. The MCL-1 inhibitor-sensitive models (DF68, DF20, DF86, DF149, and DF83) exhibited an 80–97% reduction in cell numbers, while the remaining eight PDX models were not sensitized by the addition of A-1210477 to GNE-493 (Fig. 6f, Supplementary Fig. 8D). The PDX models most sensitive to the GNE-493 and A-1210477 combination treatment were those that expressed the highest MCL-1 protein levels (Supplementary Fig. 8E), suggesting that MCL-1 represents an apoptotic vulnerability for a subset of PDX models after ‘priming’ by PI3K/mTOR inhibition. XIAP/cIAP inhibition was only effective in five PDX models (DF101, DF149, DF106, DF86 and DF20) as single-agent (Fig. 6g, Supplementary Fig. 8G). XIAP siRNA knockdown experiments mirrored the effects of treatment with birinapant on cell viability (Supplementary Fig. 8H). Combining birinapant with GNE-493 further reduced cell numbers in 9/14 PDX models by 65–94% (Fig. 6h) and drug sensitivity was predicted by the ratio of caspase-3 to XIAP protein levels, but not XIAP alone (Supplementary Fig. 8G). These results indicate that MCL-1 and XIAP contribute to cell viability of a subset of PDX models.

To determine whether MCL-1 or XIAP limit the efficacy of the ABT-737 & GNE-493 drug combination, we examined the effects of a fixed dose of the MCL-1 inhibitor or XIAP/cIAP inhibitor on varying doses of ABT-737 and GNE-493 for the eight PDX models with a range of sensitivities to combined ABT-737 and GNE-493 (DF83 shown in Fig. 6i and others in Supplementary Fig. 9A). Overall, we found that all eight PDX models could be further sensitized by triple drug combination (Fig. 6j). We classified the PDX models as responsive to the triple drug combination based on percent of area under the dose-response curve (AUC) reduction (Supplementary Fig. 9B). The PDX models fell into three groups: a) those that were sensitized by either MCL-1 or XIAP/cIAP inhibition (DF68, DF83, DF149); b) those sensitized only by MCL-1 inhibition (DF59); or c) those sensitized only by XIAP/cIAP inhibition (DF14, DF101, DF118, DF216). Notably, MCL-1 inhibition induced significant reductions in cell number (up to 95% inhibition) in the two most resistant PDX models (DF149 and DF83) that expressed high levels of MCL-1. These results, together with those of single-agent treatment with specific BCL-2 family inhibitors, highlight the critical role of MCL-1 and BCL-X_L_ in ovarian cancer cell survival and demonstrate the utility of our integrated computational-experimental approach to understand drug resistance mechanisms and identify optimal drug combinations.

## Discussion

HGS-OvCa tumors exhibit high relapse rates and chemotherapy resistance; therefore, identification of novel therapies that can induce effective tumor cell killing remains an unmet need^43^. Here, we used a clinically relevant panel of HGS-OvCa PDX models and identified apoptotic vulnerabilities that can be targeted in combination with PI3K inhibitors to induce cell death. Through an integrated approach of proteomic profiling and computational modeling, we investigated drug response mechanisms and discovered biomarkers of sensitivity (high BIM, caspase-3, and BCL-X_L_) and resistance (high MCL-1, XIAP) to the PI3K/mTOR and BCL-2/BCL-X_L_ drug combination, which may facilitate identification of those patients most likely to respond to this treatment, as well as those who might benefit by the addition of a MCL-1 inhibitor to the regimen.

Unlike many ovarian cancer cell lines, PDX models recapitulate the genomic aberrations and chemotherapy responsiveness of their corresponding primary tumors^27, 29, 44, 45^. Our panel of 14 HGS-OvCa PDX models is derived predominantly from chemoresistant tumors, which grow orthotopically in mice and exhibit drug responses in vitro that mirror those in vivo. Importantly, the PDX models exhibit diverse responses to PI3K-therapies, and thus can be used to develop novel combination treatments, discover response biomarkers and study resistance mechanisms. By combining computational modeling with selective inhibitors, our work has revealed new mechanistic insights into the nature of the adaptive responses^46^ to PI3K/mTOR treatment and, more importantly, identified biomarkers that predict sensitivity to drug combinations, which target anti-apoptotic proteins.

**Fig. 7:**
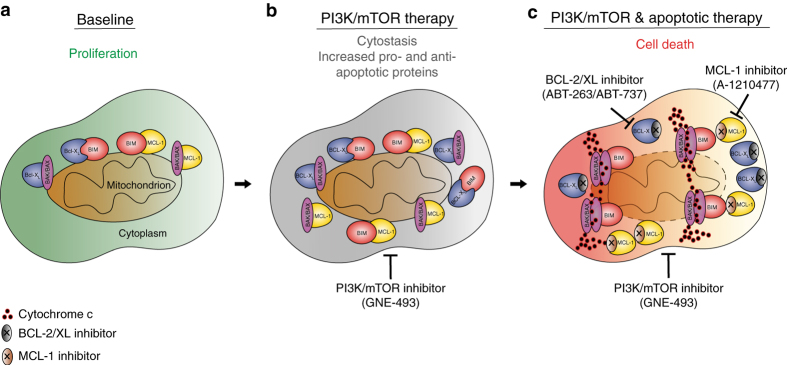
PI3K/mTOR inhibition induces elevation of both pro-apoptotic and anti-apoptotic BCL-2 family proteins that present a therapeutic vulnerability in ovarian cancer cells. **a** At baseline cells express balanced levels of the pro-apoptotic proteins BIM, BAK, and BAX and the anti-apoptotic proteins BCL-X_L_ and MCL-1. **b** Treatment with the PI3K/mTOR inhibitor results in cytostatic effects and minimal cell death due to upregulation of both pro-apoptotic and anti-apoptotic proteins that maintain a homeostatic apoptotic balance. **c** Combination treatment with an inhibitor targeting BCL-X_L_ unleashes the elevated pro-apoptotic proteins and cell death induction ensues. In ovarian cancer cells that express high MCL-1 and low BCL-X_L_, co-treatment with a MCL-1 inhibitor is required to induce cell death

Our findings are consistent with the model shown in Fig. 7, where PI3K/mTOR inhibition increases both pro- and anti-apoptotic proteins, thus maintaining a balance that is consistent with the absence of cell death. The absolute elevation in pro-apoptotic protein levels, however, “prime” cells to death upon inhibition of the coordinately increased anti-apoptotic proteins (see vector plot in Fig. 5a). These alterations in apoptosis-regulating proteins in response to PI3K/mTOR inhibition could be due, at least in part, to reversal of pro-survival signaling upon PI3K pathway blockade through inhibition of AKT-mediated pathways (e.g., phosphorylation of BAD^47^), induction of FOXO3-driven transcription of BIM/PUMA^48^, and cap-independent translation of BCL-2 family proteins upon mTOR inhibition^22^. Regardless of the mechanism of pro-apoptotic protein induction, inhibition of BCL-2 anti-apoptotic proteins in this context unleashes the activity of elevated levels of pro-apoptotic proteins and enhances cell death (Fig. 7). Dissecting the balance between pro-apoptotic and anti-apoptotic protein regulators following drug treatment using BH3 profiling^40, 49^, specific BCL-2^42, 50^ or IAP family inhibitors^51, 52^, or quantification of pro-apoptotic and anti-apoptotic proteins as in this study, can help identify drug response predictors.

Use of selective inhibitors of BCL-2, BCL-X_L_, and MCL-1 allowed us to examine the relative dependence of these proteins on pro-survival activity of the PDX tumor cells. Unlike BCL-2 inhibition, which failed to sensitize cancer cells to PI3K/mTOR treatment, BCL-X_L_ inhibition increased cell killing by GNE-493 in most of the PDX models. PDX models that exhibited poor response to the BCL-X_L_ inhibitor and high MCL-1 protein levels were sensitized to PI3K/mTOR treatment by MCL-1 inhibition. Hence, the combination of a cytostatic drug such as the PI3K/mTOR inhibitor with a BCL-X_L_ inhibitor or a MCL-1 inhibitor represents an effective combination therapy that exploits apoptotic vulnerabilities and increases cell death. MCL-1 selective inhibitors are currently in clinical trials (Clinicaltrials.gov: NCT02675452) and our findings emphasize the importance of studying BCL-2 family selective inhibitors in diverse panels of PDX models to identify effective drug combinations, coordinately with biomarkers able to predict their benefit.

In addition to the BCL-2 family apoptotic vulnerabilities, computational modeling identified the anti-apoptotic protein XIAP as a predictor of resistance to combined PI3K/mTOR and BCL-2/BCL-X_L_ inhibition. Targeting XIAP has already been proposed as a method to sensitize ovarian cancer cells to chemotherapy^51, 52^. Treatment with a XIAP/cIAP inhibitor sensitized the resistant PDX models to the PI3K/mTOR and BCL-2/BCL-X_L_ drug combination. Hence, XIAP is a promising target for further investigation, since multiple IAP inhibitors are already in Phase I and II clinical trials^53^.

We also explored the potential of combining selective PI3K, AKT and mTOR pathway inhibitors with BCL-2/BCL-X_L_ treatment, since these isoform-selective inhibitors have progressed to late stage clinical trials (Supplementary Table 4). We found that the PI3K inhibitors (GDC-0032, GDC-0941), the mTOR inhibitor (INK-128) and the AKT inhibitor (GDC-0068) could also effectively induce cytotoxic effects in combination with BCL-2/BCL-X_L_ (ABT-737) inhibition in a subset of PDX samples. Given that GDC-0032 has progressed to Phase III combination trials and ABT-263 to Phase II trials, the clinical evaluation of combined PI3K and BCL-2/BCL-X_L_ inhibition is feasible. Optimization of combination therapy dose scheduling is also a critical aspect of clinical translation. In our preclinical assessment of PI3K/mTOR therapy in combination with pulsed BCL-2/BCL-X_L_ inhibitor, we show that GNE-493 does not potentiate ABT-263 induced platelet loss and the pulsed dosing schedule allows for platelet recovery, which is an important step towards limiting on-target toxicities.

The identification of biomarkers that can predict treatment efficacy can help guide patient selection both in terms of improving outcomes and in identifying patients where additional agents in combinations may be warranted^54, 55^. Our findings indicate that high PI3K pathway activation at the protein level was associated with copy number alterations (PTEN loss, PI3KCA amplification) and predicted sensitivity to PI3K/mTOR inhibition, which is consistent with clinical trials testing PI3K/AKT/mTOR agents^13^. We also demonstrate that high BIM protein expression positively correlated with increased sensitivity to the PI3K/mTOR and BCL-2/BCL-X_L_ inhibitor combination, consistent with an ex vivo tumor slice study of single-agent ABT-737 treatment^56^. The critical role of BIM as a determinant of drug sensitivity to targeted inhibitors has been further demonstrated in other cancer types, such as lung cancer^57^ and chronic lymphocytic leukemia^58^. Detection of BIM at the RNA and protein level has been implemented in the clinic^58^, suggesting that it can be a candidate biomarker in ovarian cancer clinical trials.

Taken together, our systems approach revealed the heterogeneity of PI3K/mTOR signaling pathway activation and drug sensitivity in HGS-OvCa PDX models. PI3K-pathway inhibition induced apoptotic priming in the PDX models and targeting this apoptotic vulnerability with selective BCL-2 family inhibitors may be a promising strategy to improve treatment response in chemoresistant ovarian tumors. Furthermore, our integrated approach of drug screening, proteomic analysis and computational modeling in PDX models provides a useful framework for the rational design of personalized combination therapies and the identification of predictive biomarkers in HGS-OvCa. Given the lack of recurrent actionable point mutations in HGS-OvCa, the efficacy of this drug combination has important implications for targeted therapy development.

## Methods

### Ovarian cancer PDX models

With the exception of DF09 and DF20 which were derived from therapy-naïve patients, the primary ovarian tumors used for the generation of the PDX models were isolated from women with advanced high-grade serous ovarian cancer that have been treated with multiple chemotherapies. The establishment of these models, their clinical and genomic characteristics (e.g., BRCA mutations) are described in ref. ^34^. mCherry–Luciferase was transduced into dissociated tumor cells in vitro with a lentiviral vector and then placed under puromycin selection for 5–7 days before expansion in vivo (up to six passages) to generate viably frozen stocks. PDX models tested negative for mycoplasma contamination and were authenticated by genomic comparison with the original tumor^34^.

### Short-term in vitro experiments and cell death analysis

PDX cells were treated for 96 h in MCD105/M199 medium + 2% HIFBS + 1% Pen/Strep with vehicle or the indicated doses of drugs and luciferase signal was measured as a readout of cell numbers (Supplementary Fig. 1A). A plate with identical conditions was analyzed in parallel using a laser scanning cytometer (TTP Labtech) where dead cells were identified by staining with NuncGreen (Invitrogen). Measurement of the area under the curve (AUC) and IC_50_ values for each drug was performed using Prism (GraphPad). Drug synergy assessment was performed using the Chou–Talalay method^41^. Detailed information on the culture conditions (Supplementary Table 6), drug treatment (Supplementary Table 7) and analysis is provided in the Supplementary Methods.

### In vivo efficacy experiments

All mouse studies were conducted through Institutional Animal Care and Use Committee (IACUC)-approved animal protocols in accordance with Harvard Medical School institutional guidelines. Female NSG mice (8–10 week-old, Jackson labs) were injected intraperitoneally with 5 × 10^6^ cells. Tumor burden was monitored using BLI 1–3 weeks after tumor cell injections. Mice were randomized based on BLI into groups of five (*n* = 5, power analysis of pilot experiments using PDX model DF216 to detect differences between single-agent and combination treatment arms, power 0.8, *α* = 0.05, two-sample comparison) and were treated by oral gavage daily with GNE-493 (7 mg kg^−1^), or ABT-263 (70 mg kg^−1^) on days 4–7 and 11–14 or the combination of these drugs at the same doses as their single doses. Tumor-growth rate analysis under different treatment arms was blinded. For proteomic analysis, ascites tumor cells were harvested 2 h after the last dose, lysed with RBC buffer (Biolegend) and snap-frozen. Solid tumors and ascites cells were also formalin fixed for immunohistochemical analysis. Additional information is provided in the Supplementary Methods.

### Analysis of protein expression and computational modeling

Protein lysates were extracted from short-term in vitro and orthotopic in vivo PDX samples and RPPA arrays were performed. Western blotting was performed on the same lysates and on the independent ovarian cell line panel for validation purposes (see Supplementary Methods for more information and Supplementary Figures 10 and 11 for uncropped blots). Univariate and multivariate PLSR analyses of protein expression levels against drug sensitivity (IC_50_) were performed using a custom code in MATLAB (Mathworks). More information is provided in the Supplementary Methods.

### Statistical analysis

Values are reported as mean ± standard error of the mean (SEM) unless otherwise stated. For the biomarker identification, we used MATLAB and computed the Pearson correlation coefficient (*r*) and the associated *p* values in the original panel of the 14 patient-derived xenograft models. We also performed correlation analyses in the validation set of the seven, independent ovarian cancer cell lines. The normality of data was assessed in Prism (GraphPad) to inform the choice of a non-parametric or a parametric statistical test. Experiments involving two groups were analyzed using Student’s two-tailed *t* test and those involving multiple groups using the non-parametric Kruskal–Wallis test. *P* values below 0.05 were considered significant; their magnitude is given in the text and Supplementary Tables (for correlation analyses), and level of significance is marked by asterisks in the figures.

### Data availability

Reverse phase protein array data for the in vivo and in vitro experiments are included as Supplementary Data 1 and 2 and are available through figshare at the link: 10.6084/m9.figshare.5027516. Code is available upon request.

## Electronic supplementary material

Supplementary Information

Supplementary Data 1

Supplementary Data 2
