# Supplementary material for: Systems analysis of apoptotic priming in ovarian cancer identifies vulnerabilities and predictors of drug response
Source: Nat Commun. 2017 Aug 28;8:365. doi: 10.1038/s41467-017-00263-7 (PMC5573720; doi:10.1038/s41467-017-00263-7)
Supplement: Supplementary file 1 — Supplementary Information [file 41467_2017_263_MOESM1_ESM.pdf]

File Name: Supplementary Information
Description: Supplementary Figures, Supplementary Tables, Supplementary Methods and Supplementary References

File Name: Supplementary Data 1
Description: Proteins with altered expression ($p<0.05$) following PI3K/mTOR inhibition.

File Name: Supplementary Data 1
Description: RPPA data for short-term in vitro and in vivo experiments.

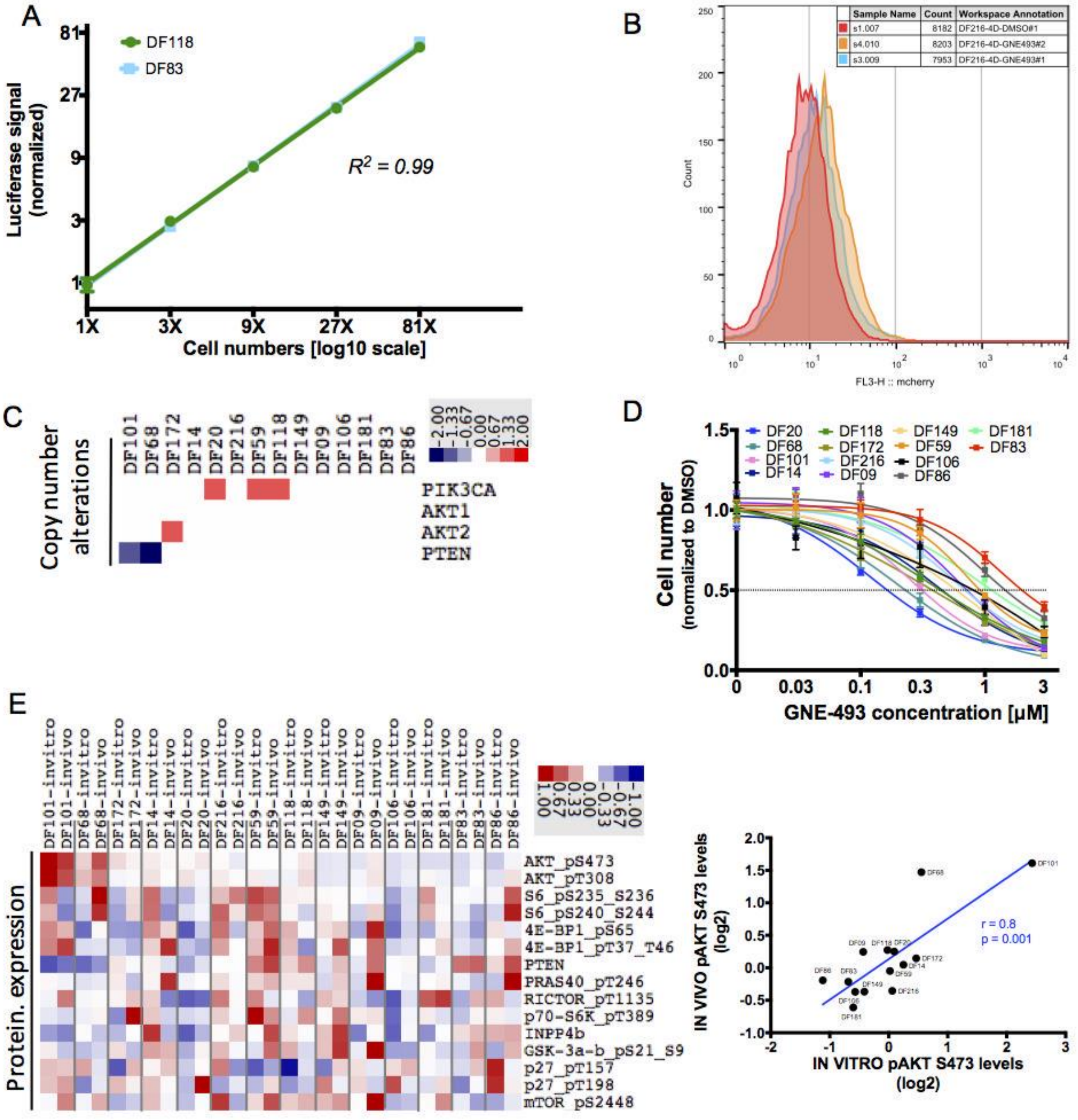

Supplementary Figure 1: Luciferase, and mCherry signals versus cell numbers, and PI3K/AKT/mTOR pathway activitation *in vitro* and *in vivo*. (A) Relationship between luciferase signal and number of cells for PDX models DF118 and DF83. **(B)** Flow analysis of mCherry expression levels for PDX model DF216 under control (DMSO, red) and GNE-493 treatment (0.5μM for 48hrs, orange/blue). **(C)** Heatmap showing copy number alterations (amplification (red) and loss (blue)) in PI3K/AKT pathway. **(D)** Luciferase-expressing HGS-OvCa cells from 14 PDX models were incubated with increasing concentrations of the PI3K/mTOR inhibitor (GNE-493) for 96 hours and assayed for luciferase signal**. (D)** Luciferase-expressing HGS-OvCa cells from 14 PDX models were incubated with increasing concentrations of the PI3K/mTOR inhibitor (GNE-493) for 96 hours and assayed for luciferase signal**. (E)** Left: Protein expression levels of PI3K/AKT/mTOR pathway targets for PDX models under short-term *in vitro* culture and *in vivo* conditions. Right: correlation of phospho-AKT^{S473} protein levels between *in vitro* and *in vivo* conditions (Pearson r=0.8, p=0.001).

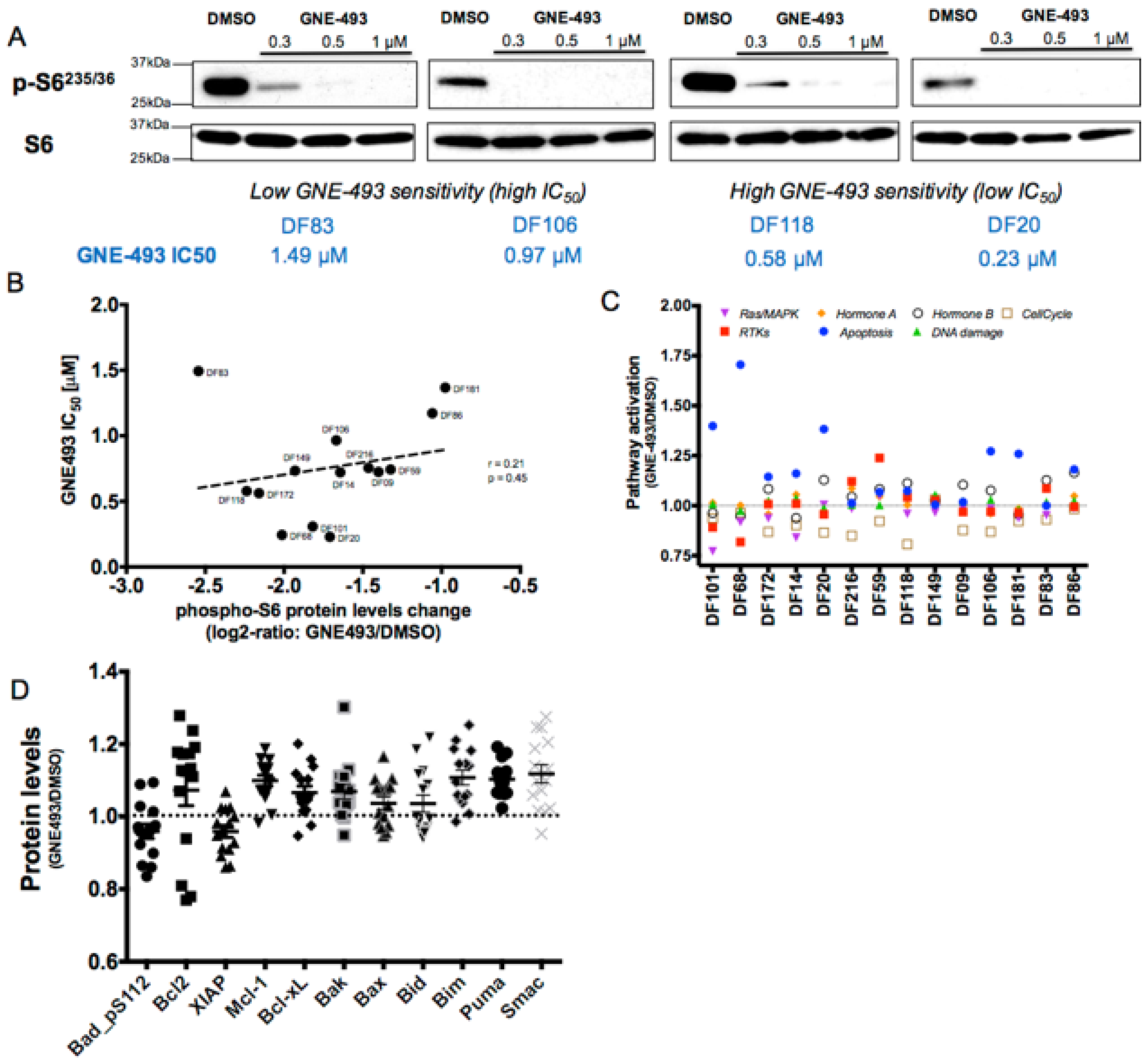

Supplementary Figure 2: Analysis of cell death, signaling pathways and apoptotic protein changes induced by GNE-493. (A) Western blot analysis for phospho-S6 protein levels after treatment with DMSO or different GNE-493 concentrations (48hr), across PDX models with different GNE-493 sensitivity **(B)** Correlation analysis of GNE-493 IC_{50} values with phospho-S6 protein level change after GNE-493 treatment (Pearson r=0.21, p=0.45). **(C)** Analysis of PI3K/mTOR pathway targets after treatment with 0.5μM GNE-493 (48hr). Each dot represents the average value for each PDX model across three replicate samples. **(C)** Pathway score ratios (GNE-493 treated over DMSO) for each PDX model. Data is derived from experiment in Fig. 2A. **(D)** Protein expression level changes (average of three replicate samples) for apoptosis-regulating proteins across all 14 PDX models. Data is representative of two independent experiments and is derived from experiment in Fig. 2D.

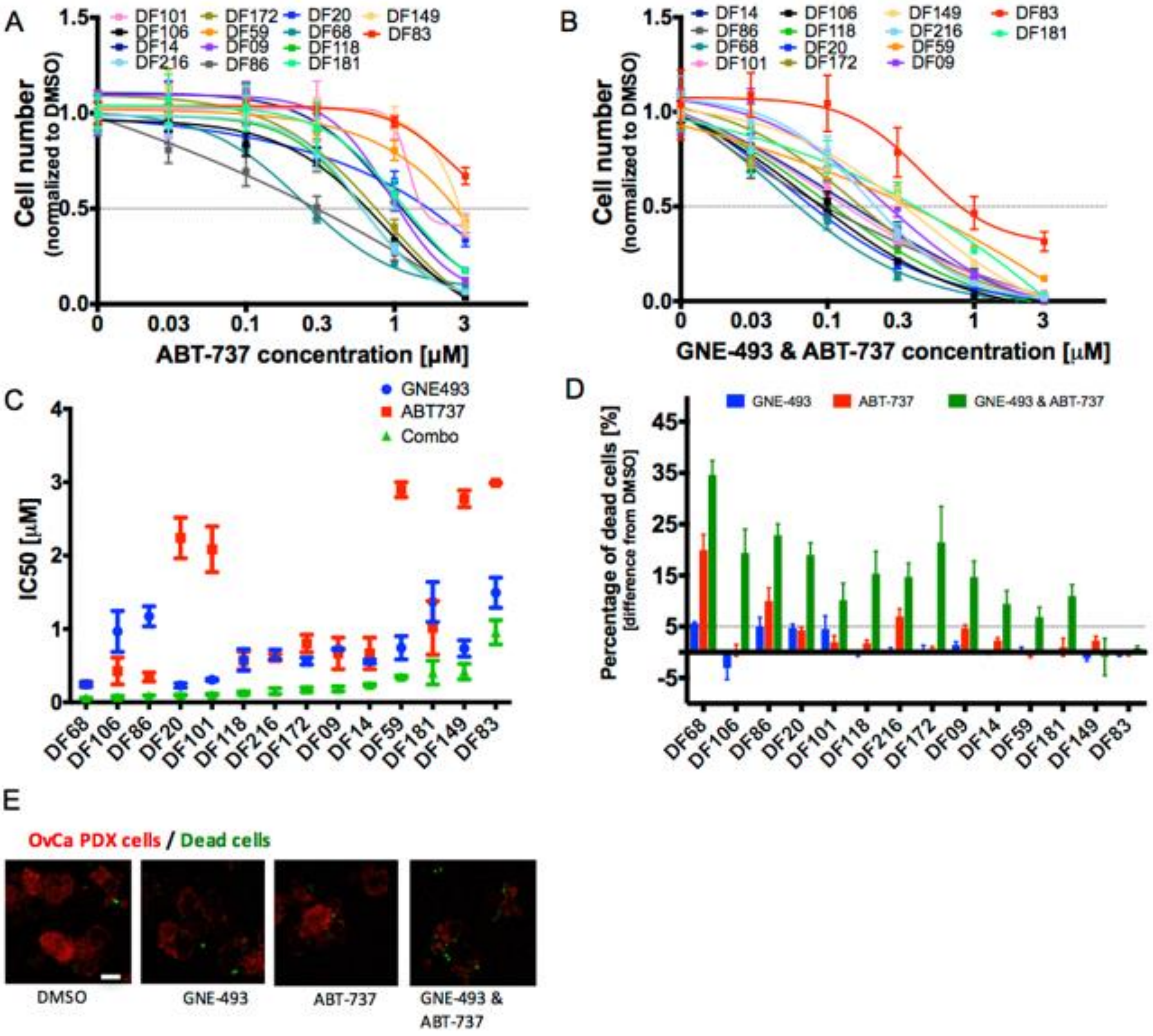

Supplementary Figure 3: Dose-response analysis for single-agent and drug combinations of PI3K/mTOR and BCL-2/BCL-X_L inhibitors. (A, B) Dose response curves for treatment with ABT-737 alone (A) and in combination with GNE-493 (B).Data is representative of three independent experiments and error bars represent SEM for n=6 replicate wells. **(C)** IC_{50} values for single agent and drug combination. Data represents the average value from three independent experiments and error bar is SEM. **(D)** Analysis of absolute cell death for single agent and drug combination (0.3µM of each drug). **(E)** Representative example of effects of drug combination (0.3µM of each drug) and single-agent on ovarian cancer cell death. DF118 PDX model (red), green: dead cells. Scale bar 50 µm.

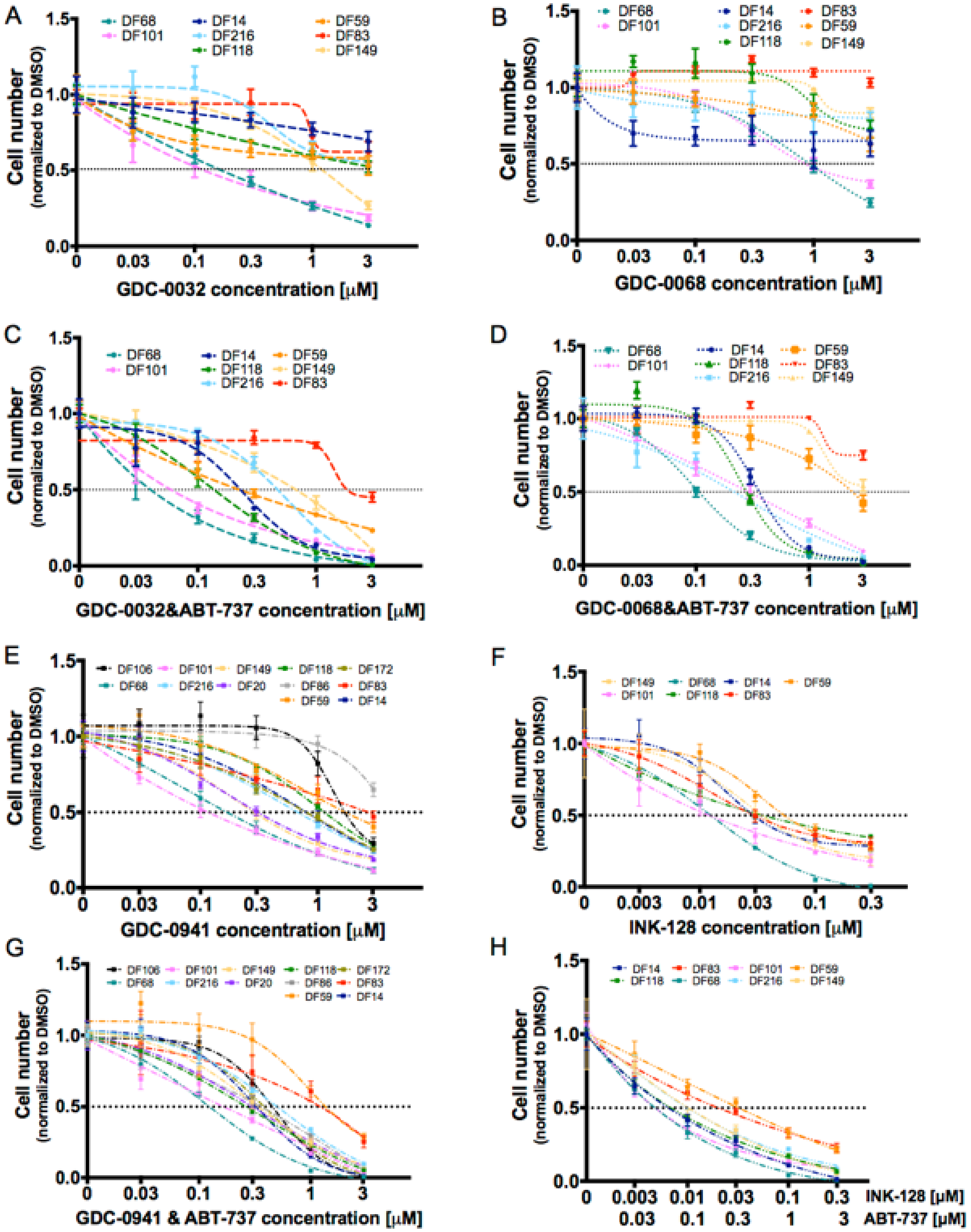

Supplementary Figure 4: Dose response analysis of PI3K/AKT/mTOR specific inhibitors and combinations with BCL-2/BCL-X_L inhibition. (A-D) Dose response curves for PI3K-specific inhibitor (GDC-0032) (A) and the AKT-specific inhibitor (GDC-0068) alone (B), or in combination with ABT-737 (C-D). **(E)** pan-PI3K inhibitor (GDC-0941) and mTORC1/2-specific inhibitor (INK-128) alone **(F)**, or in combination with ABT-737 **(G-H)**. All dose-response curves were measured 96hrs after treatment with each inhibitor or inhibitor combination. Data is representative of three independent experiments and error bars represent SEM for n=6 replicate wells.

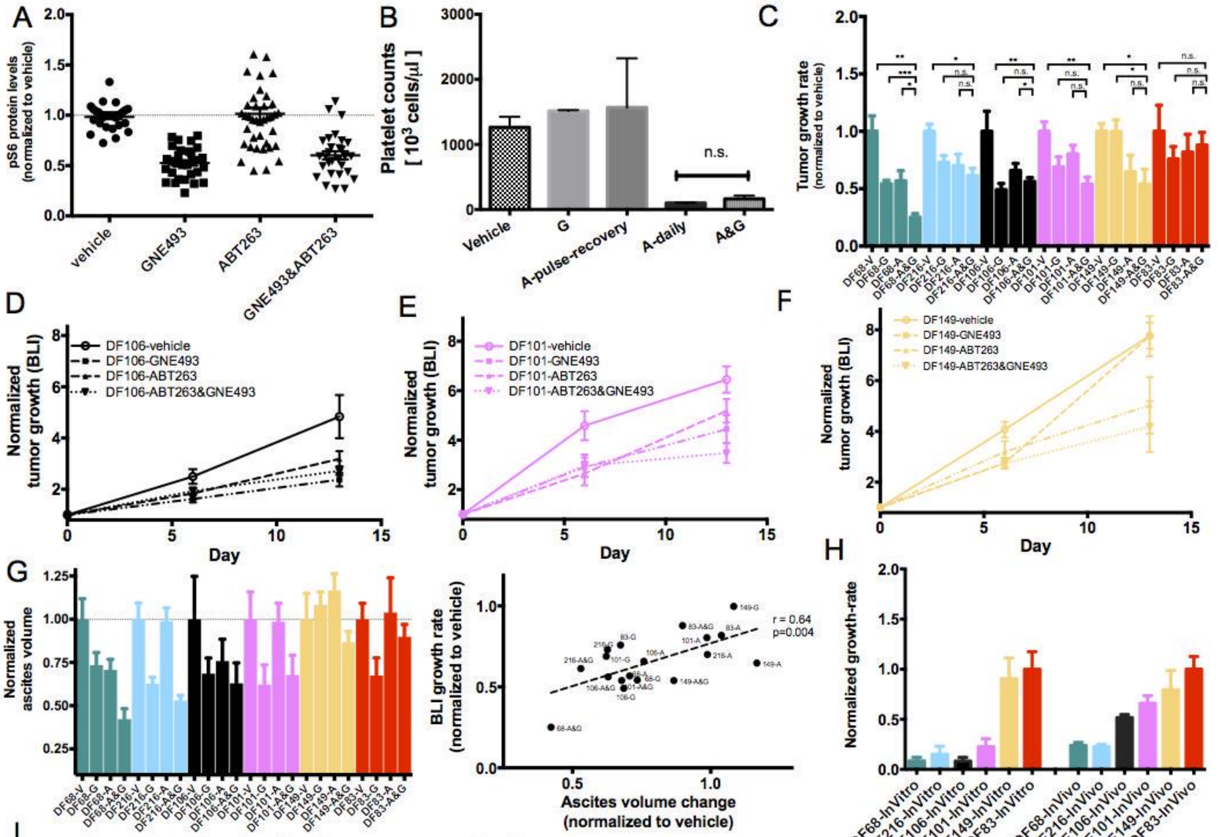

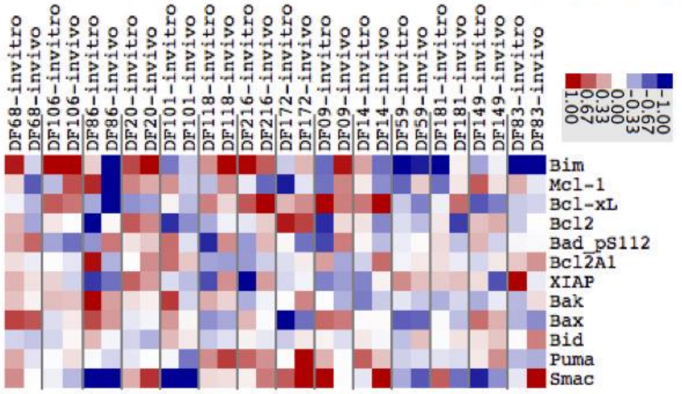

BCL-2 family

PROTEIN	Pearson (r) coefficient	p-value
BAX	0.92	0.0001
BCL-XL	0.69	0.006
BIM	0.65	0.01
MCL-1	0.44	0.12
BCL-2	0.43	0.12
PUMA	0.34	0.23
BAK	0.33	0.20
BAD_pS112	0.28	0.34
BCL2-A1	0.15	0.61
BID	0.13	0.66

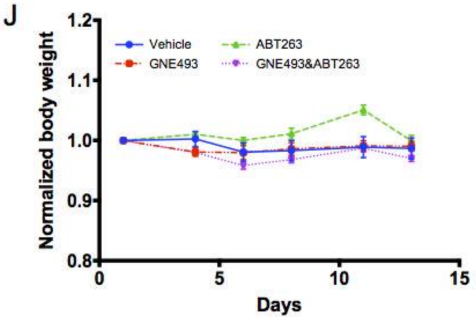

Supplementary Figure 5: Pharmacodynamic markers, tumor growth curves, ascites volume, BCL-2 family protein expression, relationship to *in vitro* drug sensitivity and animal body weight monitoring. (A) Analysis of relative phosho-S6 protein levels (normalized to vehicle) for each *in vivo* treatment arm. Each dot represents the value for one mouse (n=30 samples per treatment group: 6 PDX models x 5mice/group). **(B)** Platelet levels analysis (A corresponds to ABT-263; G to GNE-493). Values are averages for n=3 mice per treatment group and error bars are SEM. **(C)** Tumor growth rate in response to single-agent PI3K/mTOR, BCL-2/BCL-X_L and drug combination for all six PDX models determined by bioluminescence imaging. **(D-F)** Tumor growth curves for the PDX models, DF106 (D), DF101 (E) and DF149 (F) to the PI3K/mTOR (7mg kg^{-1} GNE-493) and BCL-2/BCL-X_L (70mg kg^{-1} ABT-263) drug combination. n=5 per treatment arm. Error bars are SEM. **(G)** Analysis of endpoint ascites volume (left) and correlation with bioluminescence growth-rate (Pearson r=0.64, p=0.004) **(H)** Comparison of normalized growth rates (to resistant sample DF83) under GNE-493 & ABT-263 treatment *in vitro* and *in vivo*. **(I)** Left: Protein expression levels of BCL-2 family member for PDX models under short-term *in vitro* and *in vivo* conditions. Right: correlation of BCL-2 family protein levels between *in vitro* and *in vivo* conditions. **(J)** Changes in animal body weight for each treatment group (n=5 animals per group, error bars are SEM).

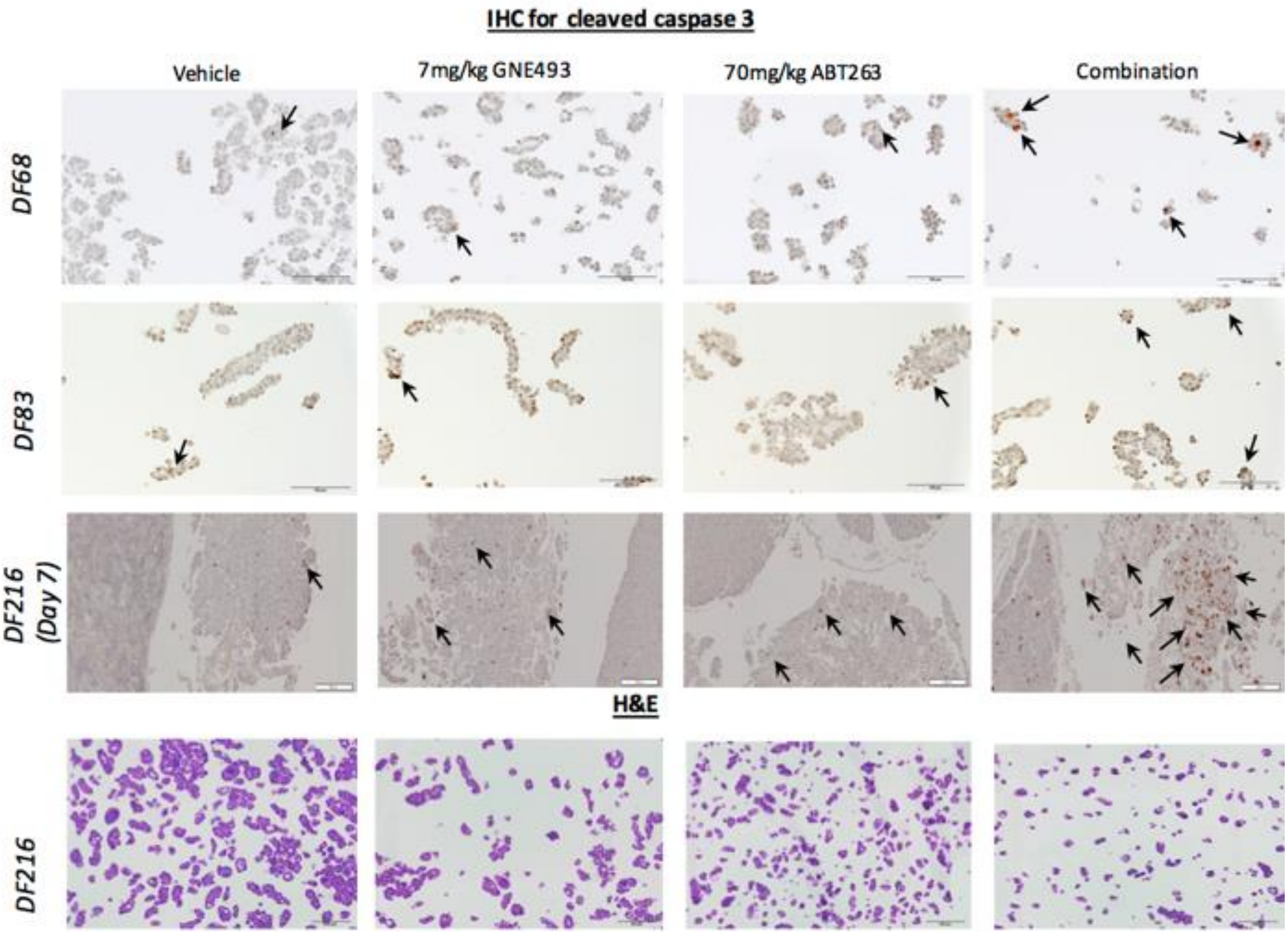

Supplementary Figure 6: Immunohistochemistry (IHC) analysis for cleaved caspase 3 staining *in vivo*. Representative cleaved-caspase-3 (cc3) IHC images from ascites tumor cells (*in vivo)* for a sensitive (DF68) and a resistant (DF83) PDX model. CC3 IHC images for sensitive PDX model DF216 seven days after treatment *in vivo.* H&E images of ascites cells (*in vivo)* for PDX model DF216. Arrows highlight cleaved caspase-3 positive cells. Scale bar 100 μm.

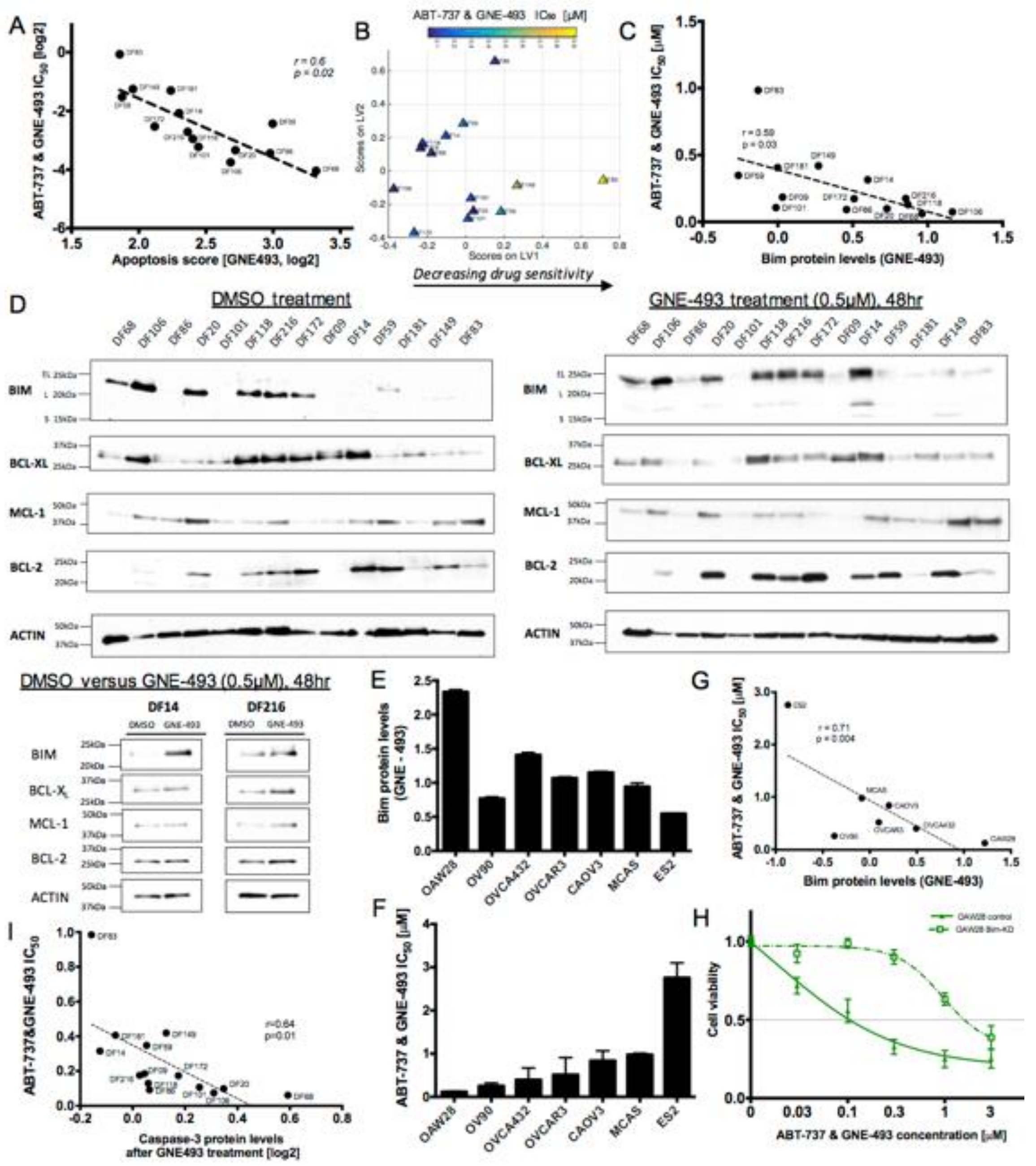

Supplementary Figure 7: Partial least squares analysis, drug sensitivity to PI3K/mTOR and BCL-2/BCL-X_L in independent ovarian cell line panel and BIM knockdown dose-response curve. (A) Correlation of apoptosis pathway score with sensitivity to PI3K/mTOR and BCL-2/BCL-X_L drug combination (Pearson r=0.6, p=0.02) **(B)** Scatterplot for partial least-squares regression (PLSR) analysis for n=2 latent variables that identified a latent variable LV1 (x-axis) that separates PDX models according to their drug sensitivity. **(C)** BIM protein levels after PI3K/mTOR inhibition in the PDX models correlate with sensitivity to the drug combination **(D)** Western blotting of BIM, BCL-2, MCL-1 and BCL-X_L protein levels from DMSO (left), GNE-493-treated (right, 0.5µM, 48hours) and matched DMSO/GNE-493 lysates (bottom left) **(E)** IC_{50} values to the GNE-493 and ABT-737 drug combination in the established cell lines. Data is representative of two independent experiments. Error bars are SEM for n=4 replicate wells. **(F)** Analysis of BIM protein levels following GNE-493 treatment at 0.5µM for 48hr by RPPA in established ovarian cancer cell lines. Data represents mean values from three replicates and error bar is SEM. **(G)** Correlation analysis of GNE-493 and ABT-737 IC_{50} values with BIM protein levels (log2-transformed) after GNE-493 treatment for the independent panel of established ovarian cancer cell lines. **(H)** Effects of BIM siRNA knockdown on OAW28 dose response to the ABT-737 and GNE-493 drug combination. Data is representative of two independent experiments. Error bars are SEM for n=4 replicate wells. **(I)** Correlation of caspase-3 protein levels after GNE-493 treatment with sensitivity to PI3K/mTOR and BCL-2/BCL-X_L drug combination (Pearson r=0.64, p=0.01).

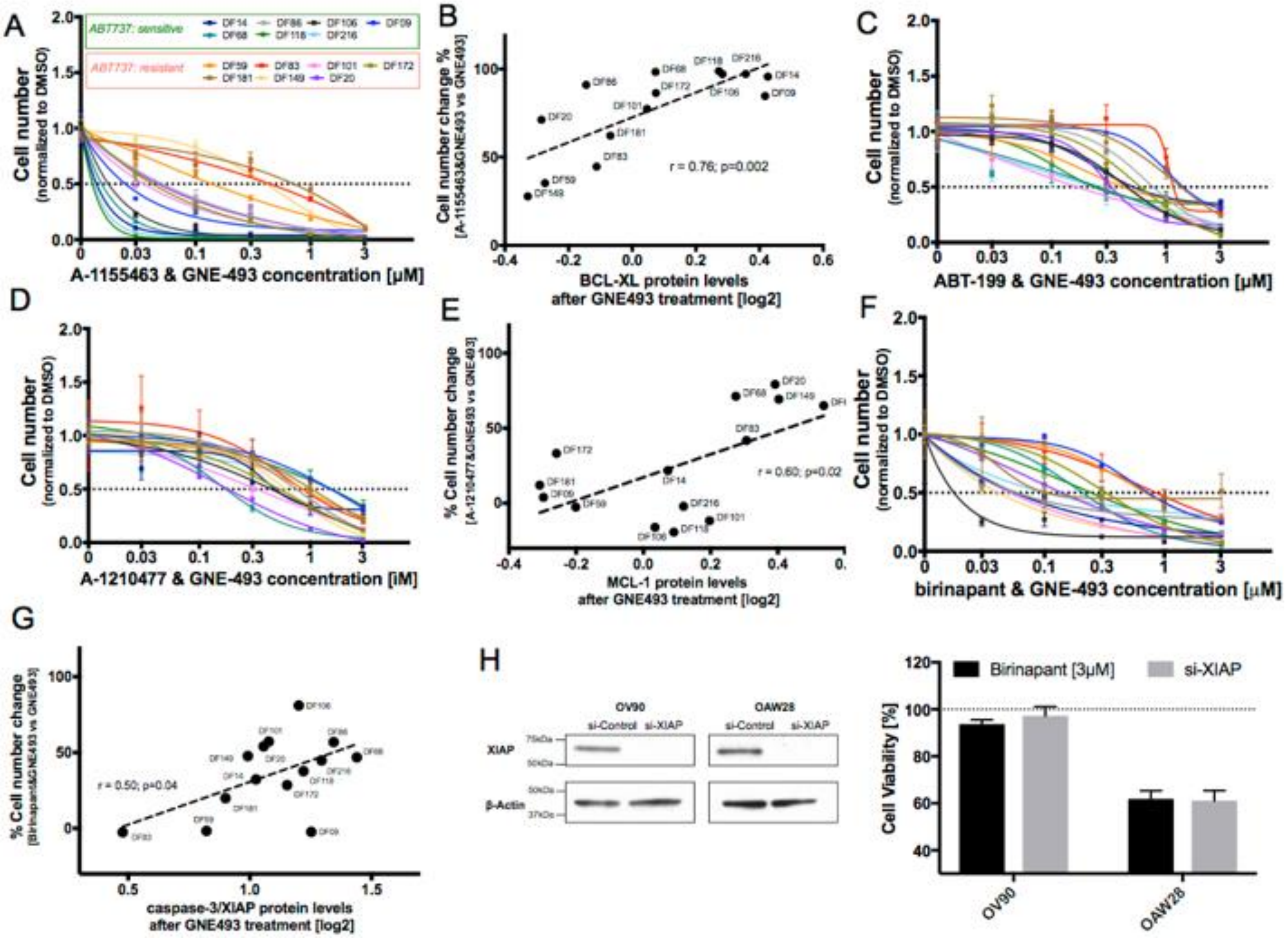

Supplementary Figure 8: Dose-response analysis of combinations of PI3K/mTOR inhibition with BCL-2/ IAP family inhibitors and XIAP siRNA knockdown. (A, C, E, F) Dose response curves for BCL-X_L-, BCL-2-, MCL-1 and XIAP/cIAP-specific inhibitors combined with GNE-493. Data is representative of three independent experiments and errors bars are SEM for n=6 replicate wells. **(B)** BCL-X_L protein levels after GNE-493 treatment correlates with sensitivity to combined GNE-493 and A-1155463 treatment. **(E)** MCL-1 protein levels after GNE-493 treatment correlates with sensitivity to combined GNE-493 and A-1210477 treatment. **(G)** Ratio of caspase-3 to XIAP protein levels after GNE-493 treatment correlates with sensitivity to combined GNE-493 and birinapant treatment. Data for (E-G) is derived from Fig. 5B). **(H)** Top: Western blot analysis of XIAP siRNA knockdown in cell lines OAW28 and OV90. Bottom: Cell viability analysis for pharmacologic (birinapant, 3μM) and genetic (XIAP siRNA knockdown) manipulation.

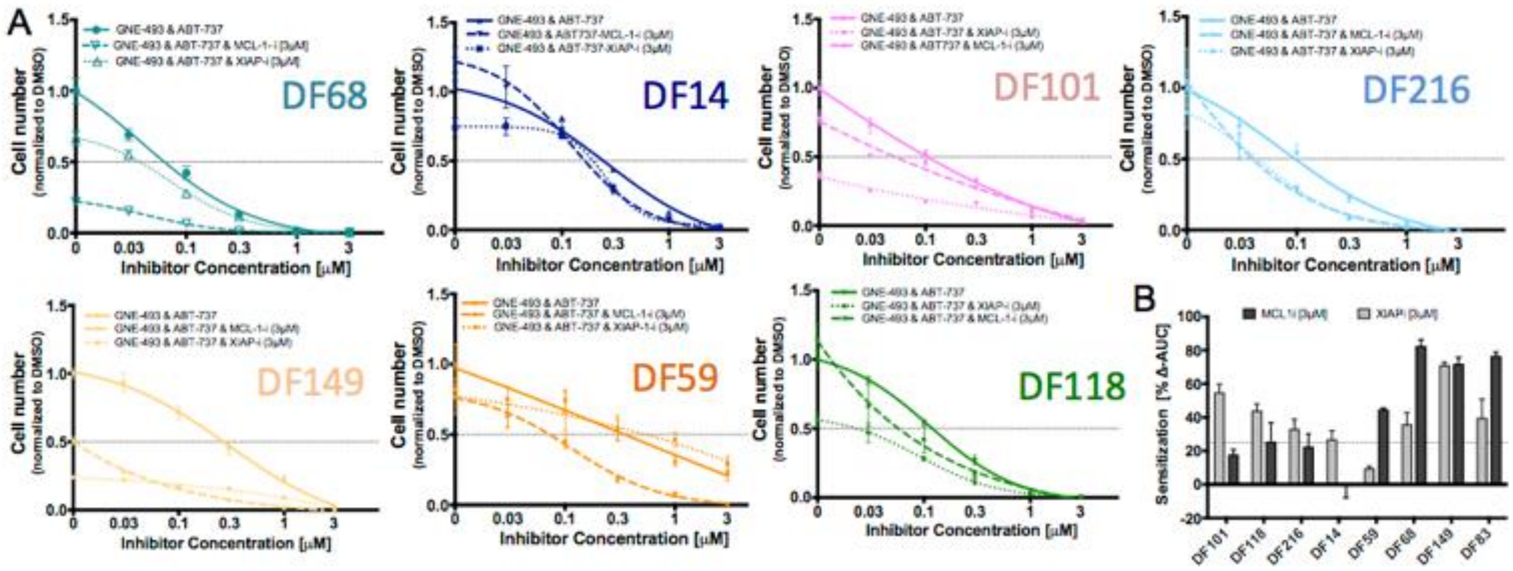

Supplementary Figure 9: Triple drug combination analysis of PI3K/mTOR inhibition with BCL-2/BCL-X_L and MCL-1 or XIAP/cIAP inhibitors. (A) Effects of MCL-1 (dashed curve) or XIAP inhibition (dotted curve) combined with ABT-737 and GNE-493 in the indicated PDX models. MCL-1 and XIAP inhibitors are present at 3μM, while ABT-737 and GNE-493 are at increasing doses in the range of 0.03-3μM. Data is representative of three independent experiments and errors bars are SEM for n=6 replicate wells. **(B)** Percent difference in area under the curve (AUC) for the triple combination therapies. Data is derived from Fig. 6I and Supplementary Fig. 9A and represents the average of three independent experiments.

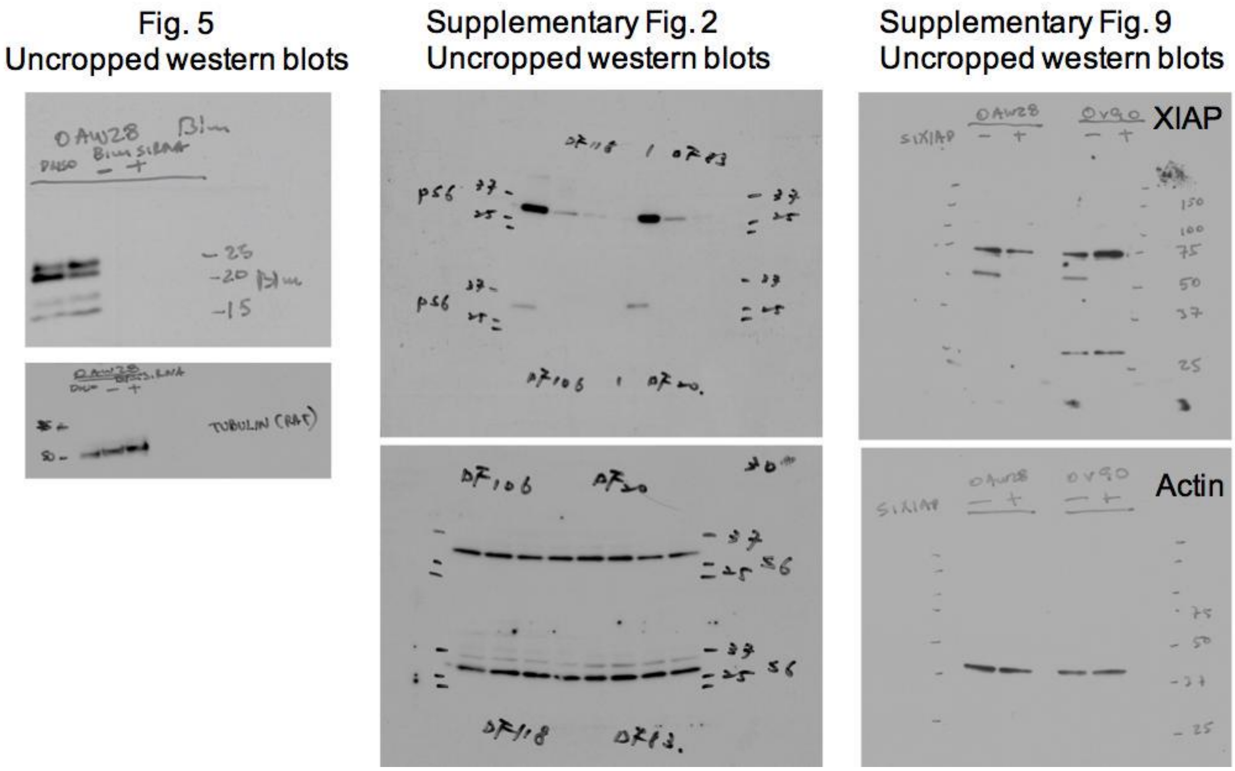

Supplementary Figure 10: Uncropped western blots from Figure 5, supplementary Figures 2 and 9.

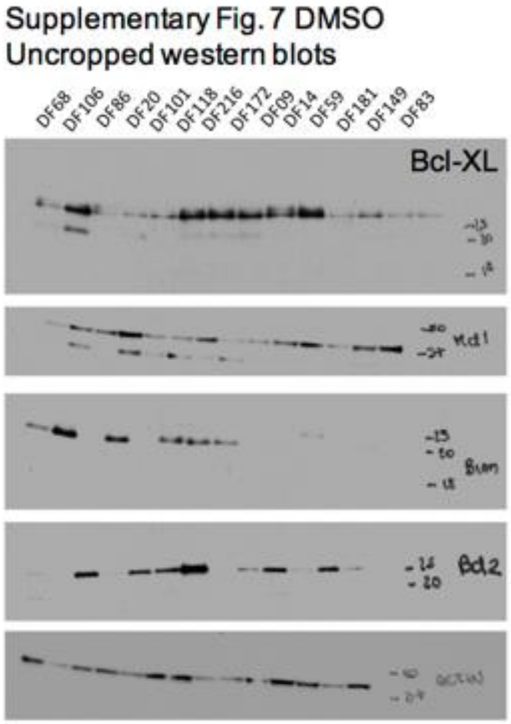

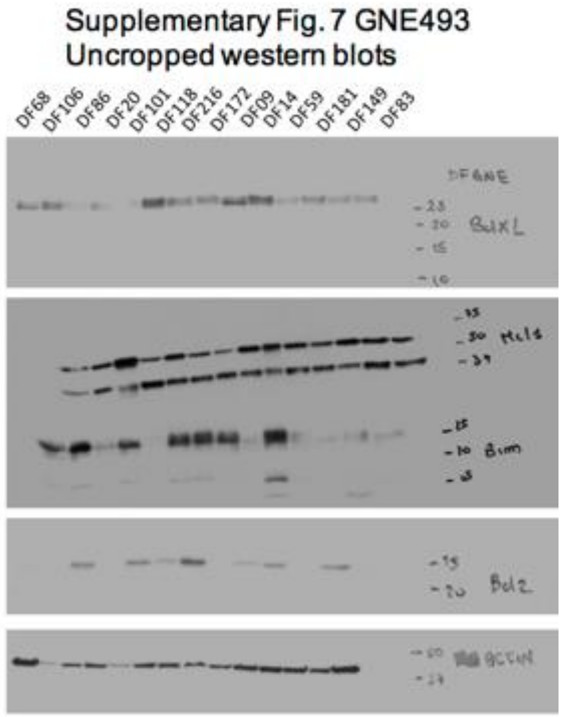

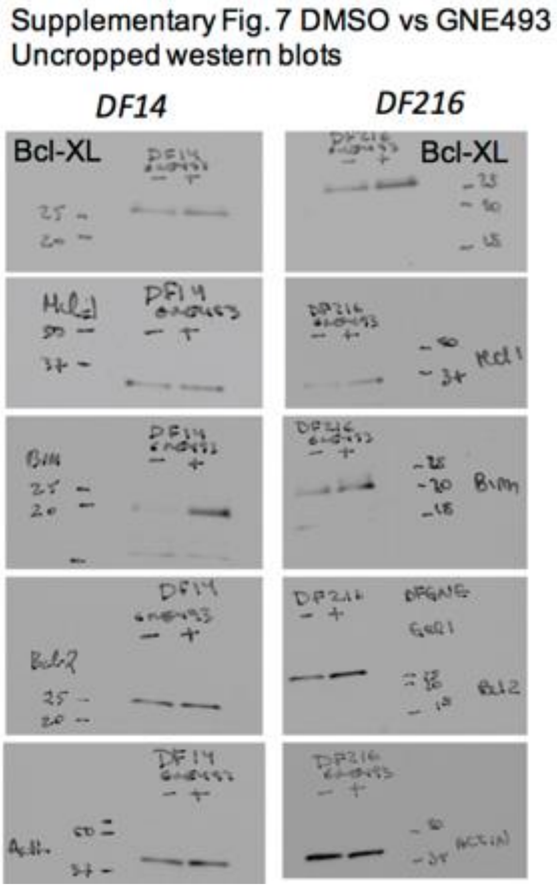

Supplementary Figure 11: Uncropped western blots from Supplementary Figure 7.

	**PI3K/Akt score (DMSO, Log2)**	**GNE493 IC50**
DF20	1.97	0.23
DF68	2.18	0.24
DF101	3.40	0.31
DF172	2.06	0.56
DF118	1.87	0.58
DF14	1.96	0.72
DF09	1.77	0.73
DF149	1.72	0.73
DF59	1.63	0.74
DF216	2.11	0.76
DF106	1.63	0.97
DF86	1.05	1.17
DF181	1.95	1.37
DF83	0.97	1.49

Supplementary Table 1. Correlation of PI3K/AKT pathway score and proteins in the PI3K/AKT/mTOR axis with GNE-493 sensitivity

Correlation of PI3K/Akt and TSC/mTOR pathway score and individual proteins with GNE493 IC50		
**Protein or pathway score**	**Pearson coefficient (r)**	**p value**
AKT_pS473	0.75	0.002
PI3K_AKT Score	0.68	0.008
PTEN	0.61	0.019
AKT_pT308	0.60	0.024
PI3K_AKT_mTOR Score	0.36	0.211
RICTOR_pT1135	0.21	0.478
S6_pS235_S236	0.19	0.507
4E-BP1_pS65	0.19	0.508
p27_pT157	0.17	0.570
p27_pT198	0.15	0.615
INPP4b	0.11	0.715
GSK-3a-b_pS21_S9	0.08	0.799
S6_pS240_S244	0.07	0.825
PRAS40_pT246	0.06	0.841
TSC_mTOR Score	0.04	0.898
4E-BP1_pT37_T46	0.03	0.918
mTOR_pS2448	0.03	0.920
p70-S6K_pT389	0.03	0.922

Supplementary Table 2. Correlation PI3K/AKT/mTOR pathway scores and proteins in the PI3K/AKT/mTOR axis with GNE-493 sensitivity

Ras/MAPK Signaling pathway	
Target	*Type of regulation*
c-Jun_pS73	+
C-Raf_pS338	+
JNK_pT183_Y185	+
MAPK_pT202_Y204	+
MEK1_pS217_S221	+
p38_pT180_Y182	+
p90RSK_pT573	+
Shc_pY317	+
YB1_pS102	+
Inhibitors in Clinical Trials: Cobimetinib, Trametinib	

PI3K/Akt Signaling pathway	
Target	*Type of regulation*
INPP4b	-
PTEN	-
Akt_pS473	+
Akt_pT308	+
GSK-3a-b_pS21_S9	+
p27_pT157	+
p27_pT198	+
PRAS40_pT246	+
Inhibitors in Clinical Trials: Tasesilib, Idelalisib, Buparlisib	

DNA Damage Signaling pathway	
Target	*Type of regulation*
53BP1	+
ATM	+
Chk1_pS345	+
Chk2_pT68	+
p53-R-C	+
Rad50	+
Rad51	+
XRCC1	+
Inhibitors in Clinical Trials: Olaparib	

RTK Signaling pathway	
Target	*Type of regulation*
EGFR_pY1068	+
EGFR_pY1173	+
HER2_pY1248	+
HER3_pY1289	+
Shc_pY317	+
Src_pY416	+
Src_pY527	+
Inhibitors in Clinical Trials: Trastuzumab, Gefitinib	

TSC/mTOR Signaling pathway	
Target	*Type of regulation*
4E-BP1_pS65	+
4E-BP1_pT37_T46	+
p70-S6K_pT389	+
mTOR_pS2448	+
Rictor_pT1135	+
S6_pS235_S236	+
S6_pS240_S244	+
Inhibitors in Clinical Trials: Everolimus, Temsirolimus	

Cell Cycle Signaling pathway	
Target	*Type of regulation*
CDK1	+
Cyclin-B1	+
Cyclin-D1	+
Cyclin-E1	+
p27_pT157	+
PCNA	+
Inhibitors in Clinical Trials: Dinaciclib, Ribociclib	

Hormone B Signaling pathway	
Target	*Type of regulation*
AR	+
INPP4b	+
GATA3	+
Bcl2	+
Inhibitors in clinical trials: Degarelix, Enzalutamide	

Apoptosis Signaling pathway	
Target	*Type of regulation*
Bad_pS112	-
Bcl-xL	-
Bcl2	-
Mcl-1	-
XIAP	-
Bak	+
Bax	+
Bid	+
Bim	+
Puma	+
Smac	+
Caspase-8	+
Caspase-3	+
Caspase-7-cleaved	+
Inhibitors in Clinical Trials: Navitoclax, Venetoclax, Birinapant	

Hormone A Signaling pathway	
Target	*Type of regulation*
ER	+
PR	+
Inhibitors in Clinical Trials: Tamoxifen	

Supplementary Table 3. Proteins (positive and negative regulators) in each pathway and drugs in trials

	Phase 2 trials	Phase 3 trials
Navitoclax (ABT263)	*Solid tumors: NCT02079740*	
	Solid metastatic tumors: NCT01989585	
	**Ovarian cancer: NCT02591095**	
	Lung cancer: NCT00445198	
	Prostate cancer: NCT01828476	
	CLL: NCT01557777, NCT00481091, NCT00918450	
	CLL: NCT01087151	
	CLL and Lymphomas: NCT00406809	
	Lymphoma: NCT01423539	
Venetoclax (ABT199)	*CLL: NCT02427451, NCT02401503*	*CLL : NCT02005471, NCT02242942*
	CLL : NCT01889186, NCT02141282	
	Lymphoma: NCT02558816, NCT02187861, NCT02471391	
	AML: NCT01994837	
Birinapant (TL32711)	*Solid tumors: NCT02587962, NCT01188499*	
	CMML: NCT02147873	
	ALL: NCT01486784	
	Ovarian cancer: NCT02756130	
	**Ovarian cancer: NCT01681368**	
Sapanisertib (INK128/MLN0128/TAK228)	*Endometrial cancer: NCT02725268*	
	Thyroid Cancer: NCT02244463	
	ALL: NCT02484430	
	Liver cancer: NCT02575339	
	Lymphoma: NCT02727777	
	Prostate cancer: NCT02091531	
	Sarcoma: NCT02987959	
	Lung cancer: NCT02417701	
	Renal cell cancer: NCT02724020	
	Bladder Cancer: NCT03047213	
	Pancreatic neuroendocrine: NCT02893930	
	Breast cancer: NCT02049957	
	Breast cancer: NCT02756364, NCT02988986	
Apotisilib (GDC0980 or GNE493)	Endometrial Cancer: NCT01455493	
	Breast cancer: NCT01437566	
	Prostate cancer: NCT01485861	
Pictilisib (GDC0941)	*Breast cancer: NCT01437566, NCT01918306, NCT01740336*	
	Non-small cell lung cancer: NCT01493843	
	Glioblastoma: NCT02430363	
Tasesilib (GDC0032)	*Breast cancer: NCT02273973*	*Breast cancer: NCT02340221*
	Breast Cancer: NCT02457910	Lung cancer: NCT02154490
Ipartesib (GDC0068)	*Breast cancer: NCT02162719, NCT02301988*	
	Prostate cancer: NCT01485861	
	Gastric cancer: NCT01896531	
	Glioblastoma: NCT02430363	
Note: only Phase 2 and later studies listed in this table; *combination trials are shown in italics*		

Supplementary Table 4. Summary of clinical trials evaluating the inhibitors used in this study

	Apoptosis score absolute value (GNE493, Log2)	ABT737&GNE493 IC50
DF68	3.319	0.061
DF106	2.683	0.074
DF86	2.978	0.092
DF20	2.720	0.099
DF101	2.444	0.107
DF118	2.402	0.129
DF216	2.364	0.153
DF172	2.118	0.173
DF09	2.997	0.185
DF14	2.300	0.237
DF59	1.875	0.348
DF181	2.238	0.406
DF149	1.956	0.419
DF83	1.858	0.954
	**Apoptosis score change (GNE493/DMSO, log2)**	**ABT737&GNE493 IC50**
DF68	0.770	0.061
DF106	0.347	0.074
DF86	0.241	0.092
DF20	0.468	0.099
DF101	0.484	0.107
DF118	0.102	0.129
DF216	0.019	0.153
DF172	0.195	0.173
DF09	0.026	0.185
DF14	0.215	0.237
DF59	0.095	0.348
DF181	0.332	0.406
DF149	0.007	0.419
DF83	0.000	0.954

Supplementary Table 5. Correlation of apoptosis score (absolute value and change after GNE-493 treatment) with GNE-493 and ABT-737 sensitivity

Supplementary Methods:

Culture conditions for PDX models

Cells were expanded *in vivo* for up to two passages by orthotopic injection in NOD-SCID mice (8-10wk old) and were viably frozen for future experiments (see image below). For *in vitro* drug profiling experiments and protein lysate preparation cells were allowed to recover for four days *in vitro* in primary ovarian growth medium (see recipe in supplementary table 6 below) prior to seeding in 96well plates in minimal medium (MCD105/M199 supplemented with 2%HI-FBS and 1% Pen/Strep).

<u>Schematic of experimental procedures:</u>

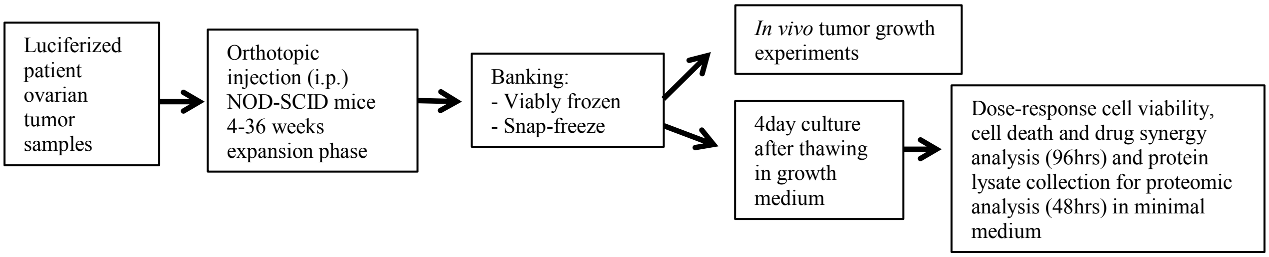

**Component**	**Volume**	**Final concentration**
Medium 199	160mL	33%
DMEM/F12	160mL	33%
RPMI-1640	160mL	33%
HI-FBS	10mL	2%
ITS	5mL	1%
Pen/strep	5mL	1%
17 beta-estradiol	10µL	0.5ng/ml
Triodothyronine	10µL	0.2pg/ml
All-trans retinoic acid	1.25µL	0.025µg/ml
Insulin [10mg/ml]	687.5µL	13.75µg/ml
Cholera Toxin [1mg/ml]	12.5µL	25ng/ml
Hydrocortisone [1mg/ml]	250µL	0.5µg/ml
EGF [100µg/ml]	50µL	10ng/ml

<u>**Supplementary Table 6. Recipe for primary ovarian cancer PDX growth medium.**</u>

Established cell lines and inhibitors

The established ovarian cell lines ES2, OVCAR3, CAOV3, OV90, OVCA432, OAW28 and MCAS were a gift from Dennis Slamon (UCLA) and were maintained in MCD105/M199 medium supplemented with 10%HI-FBS and 1% Pen/Strep (Invitrogen). The *in vitro* drug dose response experiments for these cell lines were performed as described for the PDX models.

GNE-493 [1] and GDC-0941[2] was a gift from Genentech, GDC-0032 [3], GDC-0068 [4], INK-128 [5] and birinapant [6] were purchased from Sellekchem. A-1210477 [7], A-1155463 [8], ABT-737 [9], ABT-263 [10] and ABT-199 [11] inhibitors were provided by AbbVie. Details of the compounds used in this study are provided in the supplementary table 7 below.

Compound	Target	Clinical development	Reference (PubMedID)
GNE-493	pan-PI3K and mTORC1/2	No (similar inhibitor GDC-0980 in Phase II trials)	Sutherlin DP et al (21981714) [1]
GDC-0941 (pictilisib)	pan-PI3K	Phase II trials	Folkes AJ et al (18754654) [2]
GDC-0032 (taselisib)	PI3K-α/γ/δ	Phase III trials	Ndubaku CO et al (23662903) [3]
GDC-0068 (ipatasertib)	Akt	Phase II trials	Blake JF et al (22934575) [4]
INK-128 (sapanisertib, MLN0128/TAK228)	mTORC1/2	Phase II trials	Hsieh AC et al (22367541) [5]
TL32711 (birinapant)	XIAP, cIAP1, cIAP2	Phase II trials	Allensworth et al (23225169) [6]
A-1210477	Mcl-1	No	Leverson et al (25590800) [7]
A-1155463	Bcl-XL	No	Tao et al (25313317) [8]
ABT-737	Bcl-2 and Bcl-XL	No (orally bioavailable compound ABT-263 in Phase II trials)	Oltersdorf T et al (15902208) [9]
ABT-263 (navitoclax)	Bcl-2 and Bcl-XL	Phase II trials	Tse et al (18451170) [10]
ABT-199 (venetoclax, Venclexta)	Bcl-2	FDA-approved for Chronic Lymphocytic Leukemia (April 2016)	Souers AJ T et al (23291630) [11]

Supplementary Table 7. Compounds used in this study and their targets.

Luciferase assay to measure in vitro cell numbers and flow cytometry analysis

Quantification of cell numbers *in vitro* was performed by luciferase assay and analysis of mCherry expression. The luciferase signal, which is proportional to cell numbers (see Supplementary Fig. 1A) was measured using bioluminescence imaging (Envision 3). mCherry expression, which is driven by a T2A bicistronic vector and was not affected by PI3K/mTOR inhibition (see Supplementary Fig. 1B), was quantified by flow analysis on a BD FacsCalibur instrument.

In vivo efficacy experiments

Tumor response was monitored weekly during treatment using *in vivo* bioluminescence imaging (IVIS Lumina). To monitor treatment toxicity, mice body weights were monitored every 3 days (Supplementary Fig. 5J). Blood was harvested by retroorbital bleeding, 2hours after administration of the final drug dosing, for platelet count analysis (Charles River) that served as a pharmacodynamic marker of BCL-2/BCL-X_L inhibition (Supplementary Fig. 5B). PI3K/mTOR pathway inhibition was assayed using the RPPA measurements in the ascites tumor lysates (Supplementary Fig. 5A).

Drug combination analysis

Dose-response experiments were performed for each drug pair (e.g. GNE-493 and ABT-737) as single-agents and their combination using six-point dose response curves (three-fold dilutions with a concentration range of 0.03 - 3μM and a 1:1 ratio for the drug combination experiments for all drug pairs, except for INK-128 and ABT-737 that were dosed at a 1:10 ratio). This concentration range was selected to match the reported PI3K/mTOR and BCL-2/BCL-X_L inhibitor concentrations in Phase I trials [12, 13]. The combination index (CI) for each PDX model for the ABT-737 and GNE-493 drug combination was calculated using the following formula. Dose-response curves were generated in Graphpad Prism by plotting the relative cell number (normalized by the DMSO treated condition 96hour after drug treatment) for each drug concentration. Each IC_{50} value was identified using Graphpad Prism by fitting a four parameter, variable slope dose-response curve and the CI was reported as an average of three-independent experiments.

$$CI = \frac{IC50_{ABT-737\&GNE-493}}{IC50_{ABT-737}} + \frac{IC50_{ABT-737\&GNE-493}}{IC50_{GNE-493}}$$

Partial least squares modeling

Partial least squares regression modeling was performed in MATLAB using a custom script. Z-scored protein values were used as input for the protein (x-variable) and the IC_{50} value for the ABT-737&GNE-493 drug combination was used as the y-variable. Two latent variables were used and a 45° orthogonal rotation in the LV1-LV2 plane was performed to identify a new LV1 score that strongly predicted drug sensitivity (Fig. 5C). To compute the confidence intervals for the regression coefficients of the LV1 loadings (Fig. 5D), we performed bootstrapping by leaving out one of 14 PDX models. The predictions of the partial least square regression analyses were tested experimentally using si-RNA knockdown (BIM and XIAP) and selective inhibitors (BCL-XL, BCL-2, MCL-1).

siRNA experiments

OAW28 and OV90 cells were transfected twice, first at day0 during seeding and again at day1 using Smartpool siRNA (control, BIM or XIAP, Dharmacon). On the following day, cells were treated with DMSO for 48hrs and lysates were harvested to assess transfection efficiency. Parallel dose-response experiments were conducted in 96-well plates as described above.

Reverse Phase Protein Array (RPPA) methodology, protein pathway score and copy number variation analysis

For the RPPA assay, 40 μg of protein lysate per sample was analyzed. The list of measured proteins included 288 unique antibodies (MD Anderson RPPA Core) that have been validated via immunobloting on complete gels. To adjust for protein loading variations, a normalization algorithm was applied that was based on the total protein expression levels for all antibodies analyzed. The RPPA values reported here represent relative protein levels for each

antibody normalized by the total protein content in each sample that were determined by interpolation of dilution curves from a standard calibration curve, as previously described [14].

Pathway scores were computed for nine pathways with their members listed in Supplementary Table 2. A custom script in MATLAB was used and pathway scores were computed as the sum of the relative protein levels of the positively regulated pathway members minus the negatively regulated pathway members. For the analysis presented in Fig. 2B, one-sample, two-sided t-tests were performed in MATLAB to assess statistical significance of protein expression alteration.

We performed copy number variation analysis for the PI3K/AKT pathway, by quantifying alterations in *AKT1, AKT2, PIK3CA and PTEN.* Copy number values were reported as ratios of the PDX sample to a reference normal value and were log-2 transformed for further analysis (Supplementary Fig. 1C).

Immunohistochemistry analysis

Immunohistochemisty (IHC) for cleaved caspase-3 (cc3) was carried out using paraffin sections of formalin-fixed tissue by deparaffinization, treatment with antigen retrieval buffer (Citrate pH6, Sigma) and incubation with anti-cc3 primary antibody (Cell Signaling Technology, CST 9661). Bound antibody was detected using a secondary anti-rabbit (SignalStain Boost, Cell Signaling Technology) followed by 3,3'-Diaminobenzidine staining (DAB, Sigma) and sections were counterstained with hematoxylin. A custom ImageJ script was written to automatically identify the percent of cc3-positive tumor cells (flow-chart shown in the image below). We used DAB color deconvolution to generate two images: a) an image corresponding to hematoxylin (nuclei count) and a matched image corresponding to cc3-positive cells (DAB). Cell death index was calculated as the ratio of cc3+ area over nuclei area. We analyzed at least n=30 locations (500 μm x 500 μm) per PDX model per treatment arm and selected areas distally from mouse tissue to selectively analyze human tumor cells.

<ins>Flow-chart of Image Analysis Procedure for Cleaved-caspase-3 (cc3):</ins>

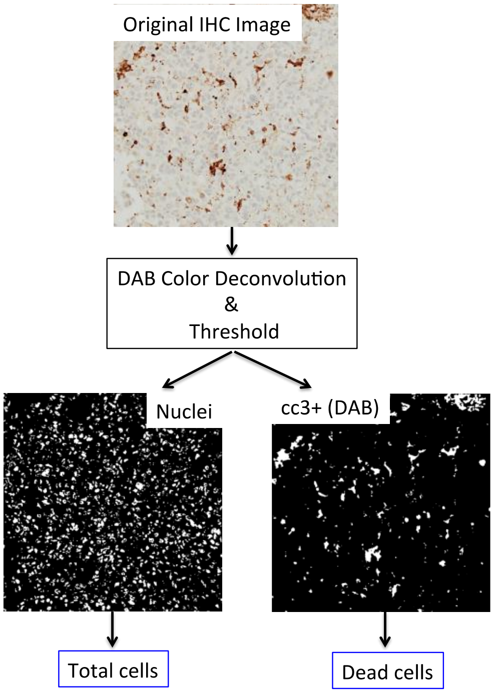

Confocal and histological image acquisition

We acquired confocal image stacks with a Nikon A1R laser scanning microscope and a 20X (NA=0.75) objective to visualize tumor cell morphology and cell death after treatment with single-agents and drug combinations. Histology slides were scanned using a semi-automated Olympus VSI120 slide scanner and a 20X objective.

Western blotting

The identical samples that were run in the RPPA assay were also analyzed by western blotting. Protein lysates were centrifuged at 13 000 rpm for 10min at 4C and supernatants were collected and assayed for protein concentration using the Pierce BCA Protein Assay Kit (Thermoscientific). Clarified lysates were boiled in 1X sample buffer for 5minutes and thirty micrograms of lysate were loaded on 4 to 12% bis-tris gel and electrophoretically transferred to PVDF membranes. Membranes were blocked for 1hr and incubated with antibodies (BIM (CST 2933S), 1/1000 dilution; BCL-2 (Abcam ab32124), 1/1000 dilution; BCL-X_L (CST 2764SS), 1/1000 dilution; MCL-1 (CST 5453S), 1/1000 dilution; XIAP (CST 2042), 1/1000 dilution; phospho-$S6^{235/236}$ (CST 4858), 1/1000 dilution; total-S6 (CST 2317), 1/1000 dilution; Actin (Sigma A1978), 1/5000 dilution; tubulin (Millipore CBL270), 1/1000) overnight. Membranes were subsequently probed with secondary antibodies linked to horseradish peroxidase prior to detection using chemiluminescent substrate and film development using a Kodak developer. Western blots were scanned using an Epson 3000 scanner.

Supplementary References

1. Sutherlin, D.P. *et al.* Discovery of (thienopyrimidin-2-yl)aminopyrimidines as potent, selective, and orally available pan-PI3-kinase and dual pan-PI3-kinase/mTOR inhibitors for the treatment of cancer. *Journal of medicinal chemistry* **53**, 1086-1097 (2010).
2. Folkes, A.J. *et al.* The identification of 2-(1H-indazol-4-yl)-6-(4-methanesulfonyl-piperazin-1-ylmethyl)-4-morpholin-4-yl-t hieno[3,2-d]pyrimidine (GDC-0941) as a potent, selective, orally bioavailable inhibitor of class I PI3 kinase for the treatment of cancer. *Journal of medicinal chemistry* **51**, 5522-5532 (2008).
3. Ndubaku, C.O. *et al.* Discovery of 2-{3-[2-(1-isopropyl-3-methyl-1H-1,2-4-triazol-5-yl)-5,6-dihydrobenzo[f]imidazo[1 ,2-d][1,4]oxazepin-9-yl]-1H-pyrazol-1-yl}-2-methylpropanamide (GDC-0032): a beta-sparing phosphoinositide 3-kinase inhibitor with high unbound exposure and robust in vivo antitumor activity. *Journal of medicinal chemistry* **56**, 4597-4610 (2013).
4. Blake, J.F. *et al.* Discovery and preclinical pharmacology of a selective ATP-competitive Akt inhibitor (GDC-0068) for the treatment of human tumors. *Journal of medicinal chemistry* **55**, 8110-8127 (2012).
5. Hsieh, A.C. *et al.* The translational landscape of mTOR signalling steers cancer initiation and metastasis. *Nature* **485**, 55-61 (2012).
6. Allensworth, J.L., Sauer, S.J., Lyerly, H.K., Morse, M.A. & Devi, G.R. Smac mimetic Birinapant induces apoptosis and enhances TRAIL potency in inflammatory breast cancer cells in an IAP-dependent and TNF-alpha-independent mechanism. *Breast cancer research and treatment* **137**, 359-371 (2013).
7. Leverson, J.D. *et al.* Potent and selective small-molecule MCL-1 inhibitors demonstrate on-target cancer cell killing activity as single agents and in combination with ABT-263 (navitoclax). *Cell death & disease* **6**, e1590 (2015).
8. Tao, Z.F. *et al.* Discovery of a Potent and Selective BCL-XL Inhibitor with in Vivo Activity. *ACS medicinal chemistry letters* **5**, 1088-1093 (2014).
9. Oltersdorf, T. *et al.* An inhibitor of Bcl-2 family proteins induces regression of solid tumours. *Nature* **435**, 677-681 (2005).
10. Tse, C. *et al.* ABT-263: a potent and orally bioavailable Bcl-2 family inhibitor. *Cancer research* **68**, 3421-3428 (2008).
11. Souers, A.J. *et al.* ABT-199, a potent and selective BCL-2 inhibitor, achieves antitumor activity while sparing platelets. *Nature medicine* **19**, 202-208 (2013).
12. Rudin, C.M. *et al.* Phase II study of single-agent navitoclax (ABT-263) and biomarker correlates in patients with relapsed small cell lung cancer. *Clinical cancer research : an official journal of the American Association for Cancer Research* **18**, 3163-3169 (2012).
13. Dolly, S.O. *et al.* Phase I Study of Apitolisib (GDC-0980), Dual Phosphatidylinositol-3-Kinase and Mammalian Target of Rapamycin Kinase Inhibitor, in Patients with Advanced Solid Tumors. *Clinical cancer research : an official journal of the American Association for Cancer Research* **22**, 2874-2884 (2016).
14. Tibes, R. *et al.* Reverse phase protein array: validation of a novel proteomic technology and utility for analysis of primary leukemia specimens and hematopoietic stem cells. *Molecular cancer therapeutics* **5**, 2512-2521 (2006).
